# A Comprehensive Review of the Phenolic Compounds in *Dracocephalum* Genus (Lamiaceae) Related to Traditional Uses of the Species and Their Biological Activities

**DOI:** 10.3390/molecules30092017

**Published:** 2025-04-30

**Authors:** Izabela Weremczuk-Jeżyna, Izabela Grzegorczyk-Karolak

**Affiliations:** Department of Biology and Pharmaceutical Botany, Medical University of Lodz, 90-151 Lodz, Poland; izabela.weremczuk-jezyna@umed.lodz.pl

**Keywords:** anti-inflammatory properties, flavonoids, hydroxyled and methoxylated flavonoids, phenolic acids, Lamiaceae, lignans, traditional Chinese medicine

## Abstract

The genus *Dracocephalum* (family Lamiaceae) comprises approximately 70 species, many of which have been traditionally used in various ethnomedical systems. The plants exhibit a broad distribution across steppe, semi-deserts, deserts, and alpine zones of temperate Eurasia, with isolated endemic species occurring in North America and North Africa. The traditional medicinal uses of the *Dracocephalum* species encompass the treatment of respiratory diseases, colds and fever, gastrointestinal disorders, liver and gallbladder ailments, musculoskeletal conditions, cardiovascular diseases, diabetes, gynecological and urological disorders, as well as ailments of the ears, throat, mouth, and eyes, as well as various dermatological conditions. The plants are rich sources of polyphenolic compounds, including flavonoids and phenolic acids, which contribute to their diverse pharmacological activities. The flavonoid profile of the *Dracocephalum* species is dominated by luteolin and apigenin derivatives, supplemented by mono-, di-, tri-, tetra-, and pentamethoxylated flavones. The predominant phenolic acids are chlorogenic acid, coumaric acid, rosmarinic acid, and their derivatives. Other phenolic compounds have also been identified in the genus: anthocyanins, lignans, phenylethanoids, phenylacetamide glycosides, flavonoid alkaloids, gingerols, coumarins, furanocoumarins, and cyanogenic glucosides. Despite growing scientific interest in this genus, a comprehensive review of its polyphenolic constituents, their structures, and associated biological activities remains lacking. To bridge this gap, this review presents an analysis of the polyphenolic profile of the *Dracocephalum* species, their ethnomedicinal uses, and the latest findings on their biological potential.

## 1. Introduction

Medicinal plants have been integral to traditional remedies for millennia, serving as a natural source of bioactive compounds. Advances in science and technology have facilitated the development of innovative formulations of herbal drugs and the extraction of valuable metabolites, which hold promise for use in various therapies, either as alternatives to synthetic drugs or as complementary treatments [1]. Recent years have seen growing interest in plants that yield valuable raw materials and medicinal compounds, particularly those utilized in traditional and local therapies [1].

The Lamiaceae family comprises an exceptional diversity of medicinal species with traditional applications. Prominent genera within this family, such as *Salvia*, *Mentha*, and *Lavandula*, are well documented for their therapeutic value [2]. Another noteworthy genus in this family is *Dracocephalum*, encompassing medicinal and aromatic plants primarily used in traditional medicine.

The genus *Dracocephalum* comprises approximately 60–70 species native to the Northern Hemisphere, with a significant concentration in the temperate regions of Asia. Most species are distributed in China, with additional occurrences in Europe, Africa, and North America. The plants are perennial, and occasionally annual, and thrive in steppes, semi-arid areas, and mountainous regions at elevations of up to 5200 m above sea level [3,4].

Several *Dracocephalum* species are key components of traditional herbal formulations, particularly in the traditional medicine of the Tibetan region, China, Mongolia, India, and Pakistan [5,6,7], as well as in Iran [8]. These plants are commonly used to manage conditions such as respiratory diseases, asthma, colds and fever, stomach disorders, hypertension, and heart disease [3,5,9]. *Dracocephalum* species are also employed to alleviate pain and treat dental ailments. In addition to their medicinal applications, some species also serve as food ingredients [10,11]. The benefits of *Dracocephalum* herbal remedies are primarily appreciated within local communities, and most lack scientific documented evidence of therapeutic effectiveness [5]. However, in recent years, increasing data on their metabolites and pharmacological activities have become available [3].

Phytochemical investigations of *Dracocephalum* species have identified approximately 500 bioactive compounds. These metabolites span various phytochemical classes, including essential oil, flavonoids, phenylpropanoids, lignans, monoterpenes, diterpenoids, triterpenoids, furanocoumarins, alkaloids, quinones, anthocyanins, and stilbenes [3,12]. Among these, the phenolic compounds are particularly notable, as they significantly contribute to the biological and pharmacological properties of the genus. Studies have confirmed that *Dracocephalum* species demonstrate antioxidant, anti-inflammatory, antimicrobial, anticancer, cardioprotective, and hepatoprotective effects [3,13,14].

This review provides a comprehensive analysis of the genus *Dracocephalum*, focusing on its phenolic composition, its traditional uses, and biological activities. It includes a detailed characterization of the most widely known and widely studied species. By synthesizing recent advancements and identifying research gaps, this work aims to highlight the therapeutic potential of the *Dracocephalum* species and their prospective applications in modern medicine, thus indicating directions for future research [15,16,17].

## 2. Literature Survey Databases

This review provides an analysis of the *Dracocephalum* genus, focusing on its traditional uses and phytochemical composition, as well as data from in vitro and in vivo studies of plant extracts and pure phenolic compounds isolated from *Dracocephalum*. It also discusses the pharmacological potential of these compounds. Relevant studies were identified using the Scopus, PubMed, ScienceDirect, and Google Scholar databases, including data published between 1954 and 2024. The search used the following keywords in combination with *Dracocephalum*: morphology, geographical distribution, traditional medicine, folk medicine, secondary metabolites, bioactive metabolites, flavonoids, phenolic acids, lignans, phenylethanoids, polyphenols, anthocyanidins, in vitro and in vivo studies, toxicity, antioxidative, anti-inflammatory, anticancer, antidepressant, sedative, antispasmotic, antimicrobial, antiviral, cytotoxic, cardioprotective, hepatoprotective, and neuroprotective potential. All included articles focused on *Dracocephalum* species, and were published in international journals.

The exclusion criteria comprised publication in a language other than English, abstracts without full text, duplicate articles and dissertations, and studies on species with only a single report or those limited to local journals, even if published in English; in addition, studies that investigated the activity of metabolites that had been purchased, i.e., the compounds were reported to have been once detected in *Dracocephalum* but they were not isolated from the genus in the described study. After initial exclusions, all inclusion and exclusion criteria were re-verified due to similarities, lack of relevance, or imprecise information, resulting in a final analysis of over 170 sources.

## 3. Botanical Description of *Dracocephalum* spp.

*Dracocephalum* is a heterogeneous genus comprising species with either erect or prostrate stems. The leaves are elongated, petiolate at the stem base, and transition to short-petiolate or even sessile as they progress upward. The leaf lamina is often oblong, ovate, or lanceolate, with margins that may be entire, crenate, or serrate. Verticillasters are arranged in dense capitula, spikes, or are widely spaced. The calyx is tubular or campanulate-tubular, straight, or slightly curved. The corolla is two-lipped, with the lower lip three-lobed (the middle lobe being the largest) and the upper lip straight or slightly curved, slender at the base, and dilated at the throat. It typically contains four stamens, with the posterior two longer than the anterior pair. The flowers are usually blue-purple, with rare occurrences of white. The seeds are oblong, smooth, and sometimes mucilaginous.

While no universally accepted division of *Dracocephalum* currently exists, Chen et al. [18] propose an infrageneric classification which recognizes nine categories based on molecular phylogenetic and morphological evidence. Most of the species described in our review were included in their analysis.

The species of Clade A are widely distributed from North America to the Sino-Japanese region and Siberia, and across Central Asia and Europe. The members are generally heterogeneous with regard to their vegetative and floral features. However, all share trigonous-ovate fruits with truncate bases and straight areoles. Of the species with therapeutic potential, *D. ruyschiana* belongs to Clade A.

The species of Clade B are mainly distributed in Central Asia. Most have a distinctive posterior calyx lip, with the three teeth almost equal in size and only half as long as the posterior lip. The clade is represented in the review by *D. peregrinum*.

Clade C can be distinguished by the following combination of characters: unbranched stems, long-petiolate basal leaves, stem leaves similar to basal, but smaller with shorter petioles, dense and capitulum-like inflorescences, and broadly ovate bracts. Most of these species are distributed in alpine areas in Central Asia and the Qinghai–Tibetan Plateau and adjacent regions. The clade includes *D. rupestre*, among others. 

Clade E consists of two subclades, one of which is mainly distributed in Central and West Asia to North Africa, and the other from Central to East Asia and Siberia; the clade includes inter alia *D. moldavica*, *D. foetidum*, *D. kotschyi*, *D. subcapitatum*, and *D. heterophyllum*.

Clade H includes species distributed in the Qinghai–Tibetan Platea region. The plants are erect without basal leaves, and are characterized by pinnatipartite to pinnatisect laminae, slightly two-lipped calyces with subequal lanceolate lobes, and a corolla with an erect or bent posterior lip. The clade is represented in the review by *D. tanguticum*.

Clade I is mainly distributed in West and Central Asia and Siberia, with a few species reported in the far east of Russia. They have either cellular or reticulate fruit surfaces. The representative species in this review is *D. palmatum*. Among the species selected for our review, *D. multicaule* and *D. polychaetum* were not included in the analysis by Chen et al. [18].

Characteristic features of the above-mentioned species discussed in the review are presented in Table 1.

## 4. Traditional Uses of Some *Dracocephalum* Species

In many countries, traditional herbal medicine serves as the primary form of therapy, and traditional drugs are often used to develop new pharmaceutical preparations. The *Dracocephalum* species described in our review are key constituents of traditional herbal formulas and are practically limited to their natural ranges. These species are mainly used in the traditional medicine of the Tibetan region, in China, Mongolia, India, Kazakhstan, and Pakistan, as well as in Iran and Mexico.

One of the most frequently described species of *Dracocephalum* is *D. moldavica*, commonly known as Moldavian balm, Moldavian dragonhead, Badershboo, or Turkish Melissa (Table 2). This plant is endemic to Central Asia (Xinjiang, China) and grows naturally in Iran, Mongolia, Egypt, and Mexico. It has also been naturalized in Western and Central Europe [10,29,30]. In these regions, the plant is traditionally used as an analgesic for neuralgia, migraines, headaches, and toothaches. Additionally, it is recognized for its use in the treatment of gastric and liver disorders, as well as coughs and asthma [10,31]. In traditional Chinese medicine, *D. moldavica* has been clinically proven to be effective in treating cardiovascular diseases, coronary heart disorders, and hypertension [31,32]. It is a primary component in several clinical preparations, including Yixin Badiranjibuya Granule, Niuhuang Shisanwei, Liganhewei pills, and Qinggan Qiwei powder [3]. In Mexican ethnomedicine, *D. moldavica* is used as a tranquilizer and to relieve nervous conditions [30]. Moldavian balm is also utilized as a food ingredient and can be consumed as tea [10,33].

*D. foetidum*, locally known as Omkhii Shimeldeg and Briyangu, is used by Mongolian nomads for the treatment of oral cavity diseases, rheumatoid arthritis, and wounds. The leaves and flowers of *D. foetidum* have demonstrated antibacterial and antifungal activities. Moreover, the flowers are used as a remedy for fever and suppurative diseases. The plant is also used for washing the face and hands [7,34].

In Mongolian ethnomedicine, *D. ruyschiana* is also an important plant. This species is considered a relict that migrated from the Russian and Asian steppes to Europe during the warm and dry interglacial periods [35]. In Europe, it is a rare but widely distributed species, primarily found in the central part of the continent, with its range extending northward to Norway and Sweden and southward to the Alps, Dinaric Alps, Carpathians, and Pyrenees [36]. *D. ruyschiana* is listed under the Bern Convention and has been assessed as Least Concern (LC) in the European Red List of Vascular Plants [37]. It is considered threatened in Norway [38] and either threatened or critically endangered in most southern European mountain ranges [35,39,40,41]. The aerial parts of *D. ruyschiana* are traditionally used for the treatment of gastric ulcers, diarrhea, and rheumatoid arthritis. They are also applied for acute respiratory infections, laryngitis, and headaches [42]. In Kazakh folk medicine, this plant is used for respiratory diseases, as an antipyretic agent, for general weakness, and as a remedy to enhance potency [43].

*D. heterophyllum* is distributed in the Xinjiang region, particularly abundant in the Autonomous Prefecture of Tuckar Kyrgyzstan, the Tibetan regions of China, and Inner Mongolia [44]. This plant has several traditional names, including Ao-Ga, Ji-Mei-Qing-Bao, Zupal, Shimthigle, B. Paul (Tibetan name), and Xinjiang Uighur Mar Xan Gusi [45]. In traditional Uygur and Tibetan medicine, *D. heterophyllum* is used for the treatment of jaundice and hepatitis. The fresh crushed leaves exhibit antimicrobial properties and are applied for the healing of purulent wounds and oral cavity ulcers [46]. Additionally, a decoction of dried flowers and leaves is effective for colds [6], digestive problems, and appetite stimulation [47]. An infusion made from the aerial parts is also used for heart palpitations, neuralgia, migraines, headaches, and symptoms of catarrhal diseases [48]. *D. heterophyllum* is also recognized as a medicinal plant in northern India, where it is traditionally known as “Shimthigle”. This species inhabits regions such as Jammu, Kashmir, Himachal Pradesh, Uttaranchal, and Sikkim at altitudes of 3000–5200 m above sea level. Traditionally, the leaf extract of *D. heterophyllum* is used for treating eye ailments, including redness, irritation, and conjunctivitis [49].

In the western regions of China, the aerial parts of *D. tanguticum* (commonly known as Zhing-Yang-Gu, Ganqingqinglan, or Tangguteqinglan) are widely used as a herbal treatments. This plant is traditionally employed for the treatment of liver and stomach diseases, arthritis, and hemorrhoids [50]. It is also considered effective in combating fever, pneumonia, and flu. Additionally, *D. tanguticum* has been used in the treatment of wounds, edema, sores, and scabies [51,52].

Traditional Asian phytomedicine also makes wide use of *D. palmatum*, known as tánara ótó (тaңapa oтo) in North Yakutia (Siberia). The young shoots and flowers of *D. palmatum* serve as a chloretic remedy and are employed in the treatment of gastrointestinal tract disorders, gastropathies, and hepatitis [25]. Moreover, this species is utilized in the folk medicine of China and Tibet for the treatment of asthma and bronchitis. *D. palmatum* also acts as a diuretic agent and is used in the treatment of alcoholism. In the food industry, the plant is a component of spices and teas [25,53].

An interesting but little-known species is *D. peregrinum*, traditionally named Tekanbasjelanbas or Tikanbasgarambas. The plant is widely distributed in northern China. It is used in Kazakh folk medicine to treat colds, exhibiting anti-cough, expectorants and, to some degree, anti-asthmatic effects. It is also employed in the treatment of liver diseases [54].

*D. rupestre* is distributed throughout northern China and Korea [55,56]. The herb is an ingredient in traditional Chinese medicine, with documented pharmacological benefits including antioxidant and antitumor activities, as well as liver-protective properties. It is primarily used to treat headaches, fever, jaundice, and liver toxicity [57,58]. Additionally, *D. rupestre* is consumed as Maojian tea in the provinces of Hebei and Shanxi, China [58].

*D. kotschyi* (Badrandjboie-Dennaie or Zarrin-Giah) is an endemic aromatic plant in Iran. In folk medicine, this plant has been used to treat gastrointestinal and liver disorders, as well as to relieve pain and manage kidney complications. The aerial parts of *D. kotschyi* are traditionally used as additives to improve the taste and scent of tea and yogurt, and as a spice for meat and fish [8,11].

*D. multicaule* (Palang Moshk or Zarrin Giah) is widely distributed in the northwestern region of Iran and is used in traditional medicine as a remedy for cardiovascular diseases, including angina, hypertension, and atherosclerosis [59]. The plant is also used for the treatment of colds and gastrointestinal disorders. *D. polychaetum* is also native to Iran and grows exclusively in the Kerman province. In traditional medicine, the herb, locally named Mofaroo, is used to relieve toothache, treat colds and kidney disorders, and as a sedative. Additionally, it is applied as a carminative and is used in the treatment of digestive and liver disorders [60].

*D. subcapitatum* is considered dominant in Iran in the Mount Missinou and Mount Akhordag regions, and the people of North Khorasan province use the plant in folk medicine, primarily as an antihyperlipidemic and antidiabetic agent [61,62].

Other little-known species of *Dracocephalum* used locally as medicinal agents include *D. fructiculosum*, *D. botrytoides*, *D. forrestii*, and *D. komarovii* [3].

The most important traditional uses of *Dracocephalum* species products, along with the regions where they are used in medicine, are summarized in Table 2.

**Table 2:** Traditional uses selected *Dracocephalum* species.

Species	Traditional Name or Name of Drugs	Region of Folk Medicinal Application	Traditional Indications	References
*D. foetidum*	Omkhii Shimeldeg, Briyangu, Fetid Dragonhead, *Tsarvon-4, *Bragshun-9, *Elegnii gurgum-7, *Anar-8, *Bavu-7, *Chun-9, *Chagdar, *Sarichun, *Jonsh-21, *Zandan -8, *Briyagu-9, *Gavar-13, *Yanjima-25,	Mongolia	Fungal and bacterial skin infections, stomatitis, rheumatic edema, and fever	[7,34]
*D. heterophyllum*	Ao-Ga, Ji-Mei-Qing-Bao, Zupal, Shimthigle, B. Paul (Tibetan name), Xinjiang Uighur Mar Xan Gusi, Jizi Qingbao	Provinces of China (Sichuan, Qinghai–Tibet Plateau, Sitsang, Qinghai, Sinkiang, Xinjiang, Neimenggu Gansu), India (Jammu, Kashmir, Himachal Pradesh, Uttaranchal, and Sikkim), India (Ladakh)	Asthma, cough, hepatitis, icterohepatitis, eye ailments (e.g., redness, irritation, conjunctivitis), mouth ulcers and tooth diseases, colds, heart palpitations, neuralgia, migraine, and lack of appetite	[45,46,47,48,49,63]
*D. kotschyi*	Badrandjboie-dennaie, Zarringiah, Zarrin-Giah	Iran (Alborz mountains, north of Khorasan)	Headache and toothaches, congestion, rheumatoid arthritis, liver and stomach disease; additive to improve the taste and scent of dishes	[8,11,64,65,66]
*D. moldavica*	Moldavian balm, Moldavian dragonhead, Toronjil azul, Toronjil chino, Xiang qing lan, Yixin Badi Ran Gibuya, BadiRanjibuya, Badrashbi, Baeiranjiboya, Melissa moldavica, Badrashboo Qinggan, *Qiwei powder, *Niuhuang, *Shisanwei pill, *Liganhewei pill, *Yixin Badiranjibuya Granules	Central, Eastern, and Nordic Europe, Siberia, Mongolia, China (Gansu, Hebei, Heilongjiang, Henan, Jilin, Liaoning, Nei Mongol, Qinghai, Shaanxi, Shanxi) Mexico, Northern and Northwestern Iran, North America	Coronary heart disease, hypertension and angina, pain (neuralgia, migraine, headache, or toothache), gastric, liver disorders, hyperactivity, and nervousness	[3,10,31,33,67,68,69,70,71,72,73,74]
*D. multicaule*	Palang moshk, Zarrin giah, Zeravihi	Iran	Cardiovascular diseases: hypertension, atherosclerosis, angina, cold, gastrointestinal disorders	[59,75,76,77]
*D. palmatum*	Tánara ótó, Palmate dragonhead	North Yakutia (Siberia), Russian Far East	Gastro-intestinal tract disorders, as diuretic and choloretic remedy, alcoholism; spice and tea	[25,53,78]
*D. peregrinum*	Tekanbasjelanbas, Tikanbasgarambas	China, Kazakhstan	Cough, asthma	[54]
*D. polychaetum*	Mafaroo, Zarro, Badranjboyeh Kermani	Iran (Kerman province)	Hyperlipidemia, inflammatory and infectious diseases, cancer, oxidative stress, indigestion, colic, stomachache, and musculoskeletal pain	[60,79]
*D. rupestre*	Maojian tea	China (Liaoning, Hebei, Nei Mongol, Shanxi, Qinghai, Shanxi), Korea	Headache, fever, jaundice and hepatitis, and damp-heat	[56,80]
*D. ruyschiana*	Northern dragonhead	Russian and Asian steppes, Kazahstan, Mongolia, Europe	Gastric ulcers, laryngitis, acute respiratory infection, diarrhea, rheumatoid arthritis, and headache	[35,43,81,82]
*D. subcapitatum*		Iran (Northeastern Khorasan)	Hyperlipidemia, diabetes	[61,62]
*D. tanguticum*	Zhi yang ge, Ganqingqinglan, Tangguteqinglan	Qinghai–Tibet Plateau, western region of China	Gastritis, hepatitis and hepatitis B virus, dizziness, rheumatoid arthritis, hypoxia, ulcers, hemorrhoids, and scabies	[51,83,84,85,86]

*—name of commercial drugs.

## 5. Phenolic Compounds from *Dracocephalum* Species

Some *Dracocephalum* species have been qualitatively screened to identify the phytochemical components responsible for their ethnomedicinal properties. These plants contain bioactive compounds such as phenylethanoids, anthocyanins, lignans, monoterpens, diterpenoids, triterpenoids, furanocoumarins, alkaloids, quinones, and stilbenes [26,29,48,86,87,88,89]. However, the most frequently reported compounds in *Dracocephalum* are flavonoids and phenolic acids. Numerous studies have demonstrated that these phytochemicals are the key contributors to the biological and pharmacological activities of the *Dracocephalum* species. In addition to the traditional use of plant products in therapy, plant-derived compounds with established biological activities can play a crucial role in the discovery of novel drugs. Current analyses indicate that natural products remain the best option for the development of innovative therapeutics for various medical conditions [90].

### 5.1. Phenolic Acids

**Figure 1:**
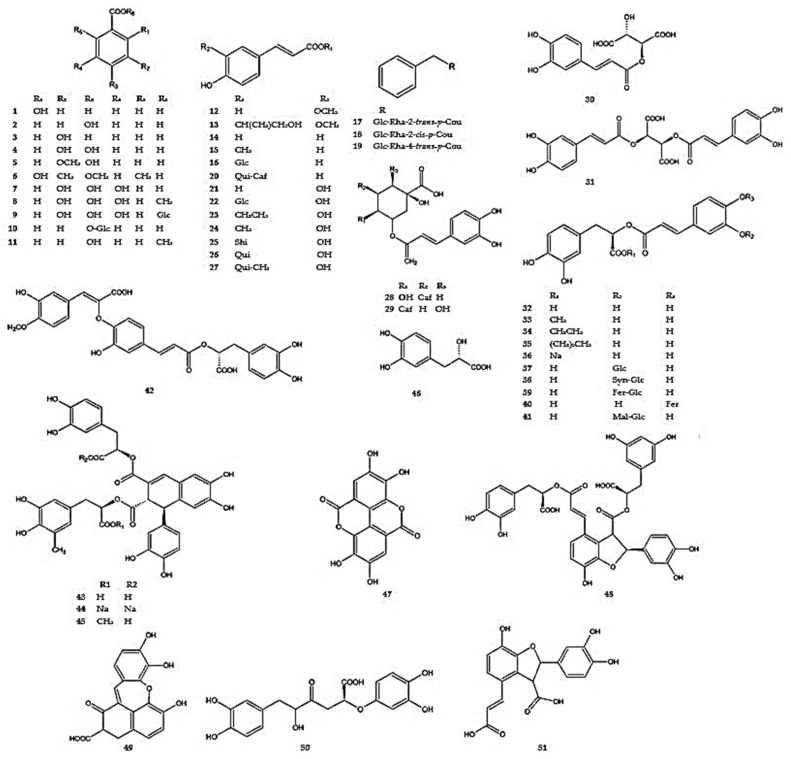
Phenolic acids and derivatives included in *Dracocephalum* species (Caf—caffeoyl, Cou—coumaryl, Fer—feruloyl, Glc—glucoside, Mal—malonyl, Qui—quinoyl, Rha—rhamnoside, Shi—shikimoyl, Syn—synapoyl).

Most of the phenolic acids in the Lamiaceae family are classified as derivatives of hydroxybenzoic or hydroxycinnamic acid [91]. Phytochemical studies of *Dracocephalum* species have identified more than fifty compounds of this type (Figure 1, Table S1).

Benzoic acids with hydroxy- and methoxy- substituents, such as 2-hydroxybenzoic acid (**1**), 4-hydroxybenzoic acid (**2**), 3-hydroxybenzoic acid (**3**), 3,4-dihydroxybenzoic acid (**4**), 3-methoxy-4-hydroxybenzoic acid (**5**), 2-hydroxy-4-methoxy-3,6-dimethylbenzoic acid (**6**), gallic acid (**7**), methylgallic acid (**8**), and *β*-glucogallin (1-galloyl-*β*-D-glucoside) (**9**), have been identified in extracts from the aerial parts of *D. heterophyllum* (**1**,**5**) [92], *D. moldavica* (**2**,**3**,**4**,**7**) [93], *D. kotschyi* (**2**,**7**), *D. polychaetum* (**2**,**7**) [13], *D. palmatum* (**2**,**8**,**9**), and *D. ruyschiana* (**6**) [26]. In an extract from *D. peregrinum*, 4-(*β*-D-glucosylo)benzoic acid (**10**) [94] was identified, while methylparaben (methyl p-hydroxybenzoate) (**11**) was identified in *D. moldavica* [29].

Ferulic acid (**12**) was detected in the aerial parts of *D. kotschyi*, *D. moldavica*, *D. polychaetum*, *D. peregrinum*, and *D. ruyschiana* [10,13,42,94], while a derivative of ferulic acid, 1′-methyl-2′-hydroxyethyl ferulate (**13**), was observed in *D. peregrinum* [94]. Additionally, p-coumaric acid (**14**) was identified in *D. kotschyi*, *D. moldavica*, *D. peregrinum*, *D. polychaetum*, *D. ruyschiana*, and *D. tanguticum* [10,13,42,94,95]. Extracts obtained from the aerial parts of *D. ruyschiana* demonstrated coumaric acid derivatives such as methyl p-coumaric acid (**15**), 1-*O*-(4-coumaroyl)-glucoside (**16**), three benzy-(coumaryl)-glucosides (**17**–**19**), and 3-p-coumaroyl-5-caffeoylquinic acid (**20**) [26,43] (Table 3).

Caffeic acid (**21**) was identified in the aerial parts of *D. moldavica*, *D. palmatum*, *D. peregrinum*, and *D. ruyschiana* [10,25,26,94]. A hexoside derivative of caffeic acid (**22**) was detected in *D. heterophyllum*, *D. ruyschiana*, and *D. palmatum* [26,87], while its ethyl and methyl esters (**23**,**24**) were identified in *D. palmatum* (**23**) and *D. peregrinum* (**24**) [26,94]. The ester of caffeic acid and shikimic acid (caffeoylshikimic acid) (**25**) was isolated from the aerial parts of *D. ruyschiana* [26].

Among the various metabolites identified in the study, the ester of caffeic acid and quinic acid, 3-*O*-caffeoylquinic acid, commonly known as chlorogenic acid, was widely distributed in *Dracocephalum* (**26**). This compound was identified in the aerial parts of *D. kotschyi*, *D. moldavica*, *D. palmatum*, *D. peregrinum*, *D. polychaetum*, and *D. ruyschiana* [10,13,25,42,94], while its methyl ester (**27**) was found in *D. tanguticum* [95]. Additionally, two dicaffeoylquinic acids were (**28**,**29**) identified in extracts from the aerial parts of *D. ruyschiana* [42].

Caftaric acid (caffeoyl tartaric acid) (**30**) and cichoric acid (dicaffeoyltartaric acid) (**31**) were reported for the first time in *Dracocephalum* plants in the herb of *D. palmatum* [25].

The ester of caffeic acid and 3-(3,4-dihydroxyphenyl)lactic acid, i.e., rosmarinic acid, is widespread in the Lamiaceae family (**32**) and was also recognized in some *Dracocephalum* species: *D. foetidum*, *D. heterophyllum*, *D. kotschyi*, *D. moldavica*, *D. palmatum*, *D. polychaetum*, and *D. tanguticum* [7,10,13,25,85,92,96]. Furthermore, three esters of rosmarinic acid, methyl rosmarinate (**33**), ethyl rosmarinate (**34**), and butyl rosmarinate (**35**), were reported in *D. foetidum* (**33**), *D. heterophyllum* (**33**,**34**), *D. moldavica* (**33**,**34**), *and D. tanguticum* (**33**,**35**) [7,92,95,97]. Additionally, sodium rosmarinate (**36**) and glucoside of rosmarinic acid (slaviaflaside) (**37**) were identified in *D. moldavica* (**36**,**37**), and *D. foetidum* (**37**) [7,69]. Several rare derivatives of rosmarinic acid, apart from rosmarinic acid glucoside, were also detected in *D. foetidum* aerial parts, including 3-(6-synapoyl-*β*-D-glucosyl)-rosmarinic acid (**38**), 3-(6-feruloyl-*β*-D-glucosyl)-rosmarinic acid (**39**), 4-*O*-(8-isoferuloyl)-rosmarinic acid (**40**), and 3-(6-malonyl-*β*-D-glucosyl)-rosmarinic acid (**41**) [7].

*D. foetidum* was found to contain a caffeic acid trimer (**42**) [7], while the tetramer, rabdosiin, was reported in *D. moldavica* (**43**), along with its sodium (**44**) and methyl derivatives (**45**) [69]. In addition, 3-(3,4-dihydroxyphenyl)-2-hydroxypropanoic acid, also known as danshensu (**46**), was isolated from an aqueous extract of *D. moldavica* [69].

Ellagic acid (**47**), a dimer of gallic acid, was described in extracts from *D. moldavica* and *D. ruyschiana* [26,98]. Other polyphenolic acids identified in *D. palmatum* include salvianolic acid B (**48**), salvianolic acid G (**49**), salvianic acid C (**50**), and prolithospermic acid (**51**) [25,26]. Additionally, salvianolic acid B was detected in *D. moldavica*, D. palmatum, and *D. tanguticum* [25,99].

### 5.2. Flavonoids

**Figure 2:**
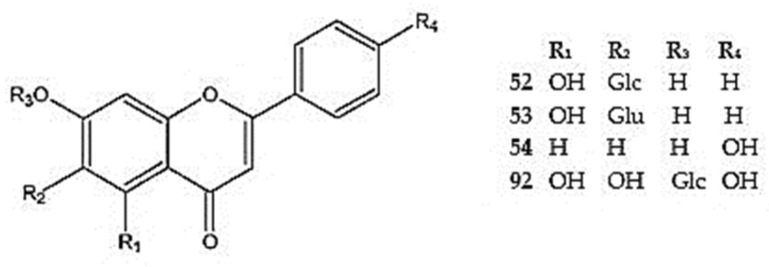
Different flavone derivatives detected in *Dracocephalum* species (Glc—glucosyl, Glu—glucuronic).

**Figure 3:**
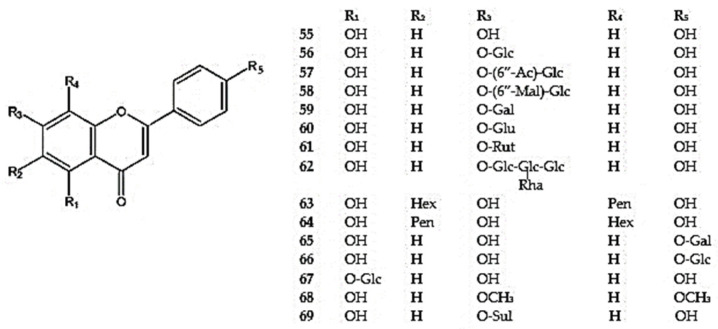
Apigenine derivatives detected in *Dracocephalum* species (Ac—acetyl, Glc—glucoside, Glu –glucuronic, Hex—hexoside, Mal—malonyl, Pen—pentoside, Rha—rhamnoside, Rut—rutoside).

**Figure 4:**
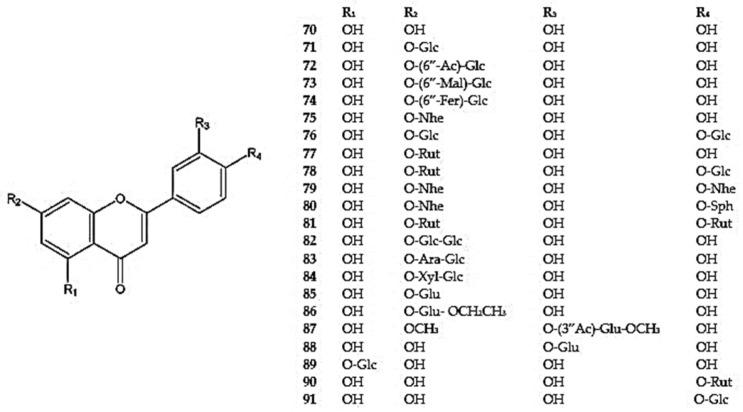
Luteoline derivatives detected in *Dracocephalum* species (Ac—acetyl, Ara—arabinoside, Glc—glucoside, Glu –glucuronic, Fer—feruosyl, Nhe—neohesperidoside, Rut—rutoside, Sph—sophoroside, Xyl—xyloside).

**Figure 5:**
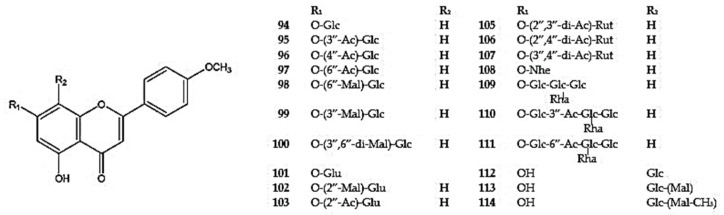
Acacetin derivatives detected in *Dracocephalum* species (Ac—acetyl, Glc—glucoside, Glu –glucuronic, Mal—malonyl, Nhe—neohesperidoside, Rha—rhamnoside, Rut—rutoside).

**Figure 6:**
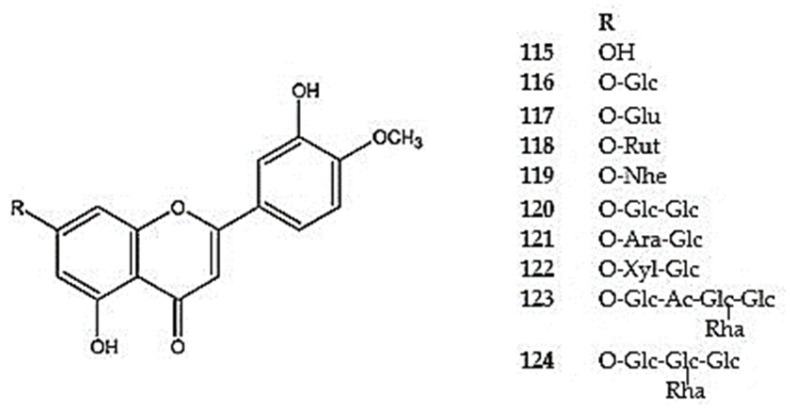
Diosmetin derivatives detected in *Dracocephalum species* (Ac—acetyl, Glc—glucoside, Glu –glucuronic, Nhe—neohesperidoside, Rut—rutoside).

**Figure 7:**
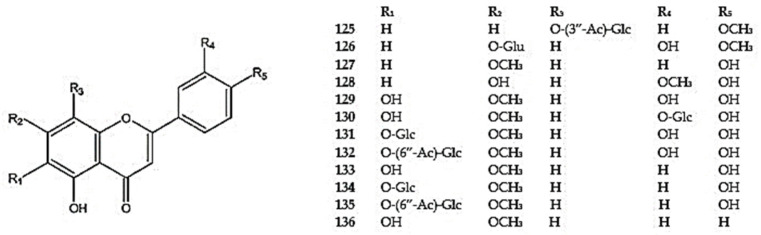
Monomethoxyflavones detected in *Dracocephalum species* (Ac—acetyl, Glc—glucoside, Glu—glucuronic).

**Figure 8:**
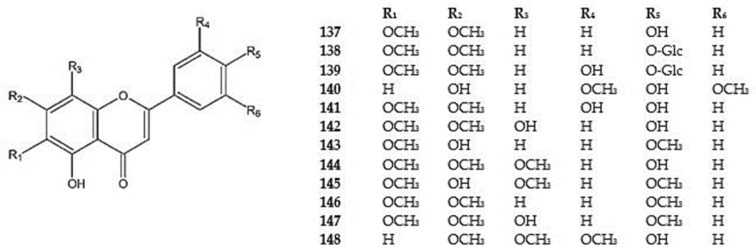
Di- and trimethoxyflavones detected in *Dracocephalum* species (Glc—glucoside).

**Figure 9:**
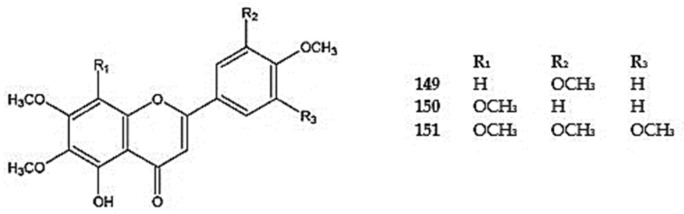
Tetr- and hexamethoxyflavone detected in *Dracocephalum* species.

**Figure 10:**
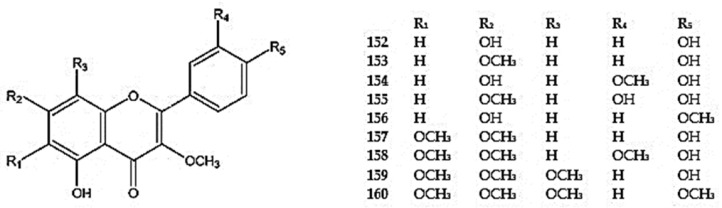
3-*O*-methoxylated flavones detected in *Dracocephalum* species.

**Figure 11:**
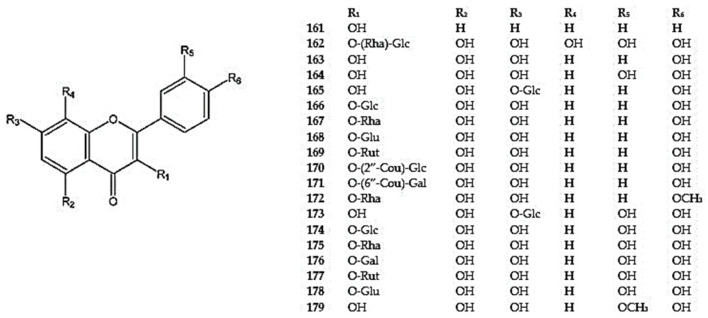
Flavonols and their sugar derivatives detected in *Dracocephalum* species (Cou—coumaryl, Gal—galactoside, Glc—glucoside, Glu –glucuronic, Rha—rhamnoside, Rut—rutoside).

**Figure 12:**
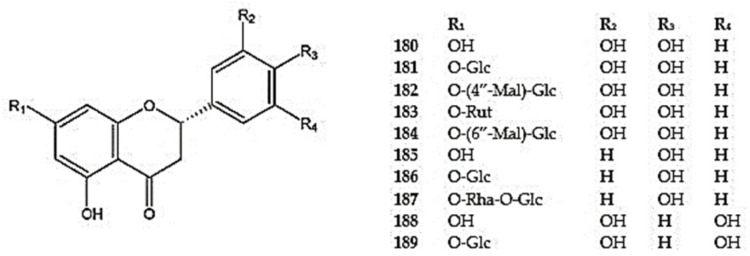
Flavanone derivatives detected in *Dracocephalum* species (Glc—glucoside, Mal—malonyl, Rha—rhamnoside, Rut—rutoside).

**Figure 13:**
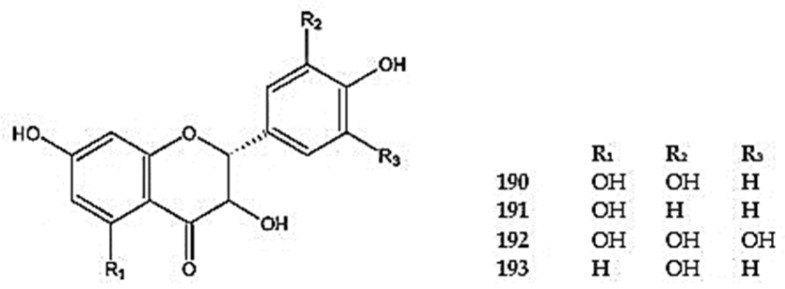
Flavanonol derivatives detected in *Dracocephalum* species.

**Figure 14:**
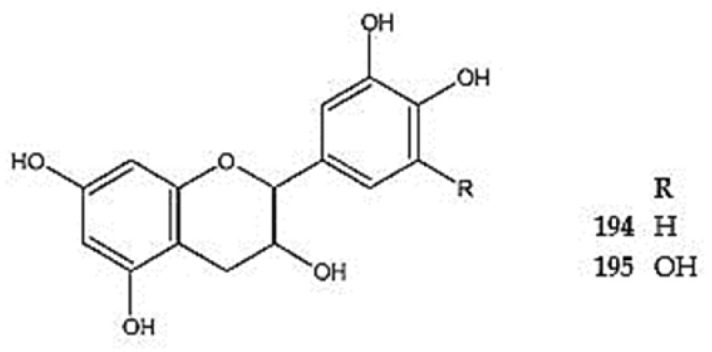
Flavan-3-ol derivatives detected in *Dracocephalum* species.

Zeng et al. [12] reported the presence of about 50 flavonoids in the genus *Dracocephalum*, among other secondary metabolites. Studies conducted over the last decade have provided more detailed information on these active compounds in the *Dracocephalum* species with medicinal uses. For the *Dracocephalum* species described in this review around 150 flavonoid compounds were reported (Figure 2, Figure 3, Figure 4, Figure 5, Figure 6, Figure 7, Figure 8, Figure 9, Figure 10, Figure 11, Figure 12, Figure 13 and Figure 14). The species in *Dracocephalum* often exhibit hydroxylated flavones, mainly luteolin, diosmetin, apigenin and acacetin, and their glycosides; these constituents, particularly with substitution types 5,7,4′ and 5,7,3′,4′, are specific to this genus [25]. Also, mono-, di-, tri-, tetra-, and pentamethoxylated flavones are characteristic to the *Dracocephalum* species [100] (Table S1).

#### 5.2.1. Flavone Derivative

Among the dihydroxyflavones, chrysin (5,7-dihydroxyflavone-6-*C*-glucoside) (**52**) and its glucuronide (**53**) were identified in *D. ruyschiana* [26], while 7,4′-dihydroxyflavone (**54**) was detected in *D. moldavica* [101] (Figure 2).

Apigenin (5,7,4′-trihydroxyflavone) (**55**), a flavone widely distributed in *Dracocephalum* and in other genera of the Lamiaceae family, and its derivatives are summarized in Figure 3. Apigenin 7-*O*-*β*-D-glucoside (**56**) has been identified in the aerial parts of *D. kotschyi*, *D. moldavica*, *D. multicaule*, *D. palmatum*, *D. rupestre*, *D. ruyschiana*, and *D. tanguticum* [10,11,25,26,55,100,102]. Derivatives of apigenin 7-*O*-*β*-D-glucoside with additional acyl and malonyl groups (**57**,**58**) at the C-6″ position were identified in *D. palmatum* (**57**,**58**) and *D. foetidum* (**58**) [7,103] (Table S1).

Apigenin-7-*O*-galactoside (**59**) was detected in an extract of *D. moldavica* [104], while apigenin-7-*O*-β-D-glucuronide (**60**) was reported in extracts from *D. moldavica*, D. *ruyschiana*, and *D. foetidum* [7,26,105]. Apigenin substituted with an *O*-(α-L-rhamnosyl)-(1→6)-glucosyl moiety at position 7 via a glycosidic linkage, named apigenin-7-*O*-rutinoside (isorhoifolin) (**61**), was identified in *D. palmatum* and *D. heterophyllum* [53,92]. Additionally, apigenin C7 polyglycoside was found in the aerial parts of *D. ruyschiana* (**62**) [42].

Apigenin C6 and C8 diglycosides (**63**,**64**) were isolated from the aerial parts of *D. ruyschiana* [26]. Furthermore, apigenin glycosides positioned at C4′ (**65**,**66**) and C5 (**67**) were found in *D. kotschyi* (**65**,**66**) [96,106], *D. palmatum* (**68**) [26], and *D. multicaule* (**67**) [100]. Other apigenin derivatives detected in the *Dracocephalum* species include apigenin-7,4′-dimethyl ether (**68**) in *D. palmatum* and apigenin-7-sulfate in *D. ruyschiana* (**69**) [26].

Luteolin (5,7,3′,4′-tetrahydroxyflavone) (**70**) is a well-known bioactive flavone. It is commonly found in the Lamiaceae family and widely distributed throughout *Dracocephalum*, together with its derivatives. They have been detected in all described species except *D. foetidum* (Table S1) (Figure 4).

The most extensive group of luteolin glycosides include a sugar moiety, connected via an oxygen atom at the C7 position. The most common glycoside is cynaroside (luteolin-7-*O*-*β*-D-glucoside) (**71**) which has been identified in *D. kotschyi*, *D. moldavica*, D. *multicaule*, *D. palmatum*, *D. peregrinum*, *D. rupestre*, and *D. tanguticum* [26,55,94,96,100,102,105]. In *Dracocephalum* species, derivatives of luteolin-7-*O*-glucosides with additional groups at the C6″ position have also been identified. These include acetyl or malonyl groups (**72,73**) in *D. palmatum* [103] and a ferulyl group in *D. peregrinum* (**74**) [94].

Luteolin substituted with *O*-*β*-rhamnosyl-(1→2)-glucoside at position 7, referred to luteolin 7-*O*-*β*-D-neohesperidoside (**75**), was found in *D. moldavica* [105] (Figure 4). Olennikov and Chirkova [107] isolated rarely occurring luteolin diglycosides and polyglycosides from the aerial parts of *D. palmatum*, including luteolin-7,4′-di-*O*-glucoside (**76**), luteolin-7-*O*-rutinoside (**77**), luteolin-7-*O*-rutinoside -4′-*O*-glucoside (**78**), and luteolin-7,4′-di-*O*-neohesperidoside (**79**). These were accompanied by luteolin substituted with neohesperidoside at position 7 and 4-*O*-*β*-glucosyl-(1→2)-glucoside at position 4′, thus forming luteolin-7-*O*-neohesperidoside-4′-*O*-sophoroside (**80**). Additionally, luteolin-7,4′-di-*O*-rutinoside (dracopalmaside) (**81**) was identified for the first time in this species [107]. Luteolin-7-*O*-rutinoside was also detected in *D. heterophyllum* and *D. peregrinum* [92,94].

Three other luteolin diglycosides, luteolin-7-*O*-*β*-D-glucosyl-D-glucoside (**82**), luteolin-7-*O*-*α*-L-arabinosyl-(1→6)-*β*-D-glucoside (**83**), and luteolin-7-*O*-*β*-D-xylosyl-(1→6)-*β*-D-glucoside (**84**), were identified in *D. tanguticum* [50], whereas luteolin-7-*O*-*β*-D-glucuronide (**85**) was found in *D. foetidum*, *D. moldavica*, and *D. ruyschiana* [7,26,105], and its ethyl ester (**86**) and the luteolin-7-methoxy-3′-*O*-(3″-*O*-acetyl)-*β*-D-glucuronide-6″-methyl ester (**87**) were identified in *D. tanguticum* [102,108] (Table S1).

Moreover, the 5, 3′, or 4′-substituted luteolin glycosides were found in *D. kotschyi* (**88**), *D. hetreophyllum* (**89**), and *D. palmatum* (**90,91**) [25,92,96,107] (Figure 4). In *D. peregrinum*, plantaginin (7-*O*-glucoside of 5,6,7,4′-tetrahydroxyflavone) (**92**) was also detected by Dai et al. [94] (Figure 2).

Several studies have demonstrated the presence of flavones containing varying numbers of methoxy and/or hydroxy groups in some *Dracocephalum* species, with these being substituted at positions 6–8 and 3′–5′ of the flavone rings. These mono-, di-, tri-, tetra-, and pentamethoxylated flavones are likely synthesized through the same chemical pathway, involving the stepwise methylation of apigenin [11].

The dominant compounds in this group include 5,7-dihydroxy-4′-methoxyflavone, acacetin (**93**), and its 7-*O*-substituted derivatives (**93-111**) (Table S1) (Figure 5). Acacetin has been identified in *D. moldavica*, *D. multicaule*, *D. palmatum*, *D. peregrinum*, and *D. ruyschiana* [26,53,100,105,109]. Among these species, the most commonly reported compound is acacetin-7-*O*-glucoside (tilianin) (**94**) [26,53,94,105]. Additionally, tilianin was detected in *D. foetidum*, *D. kotschyi*, and *D. tanguticum* [7,96,102]. Several acylated and malonyled acacetin-7-*O*-glucosides have also been isolated. Acetylated derivatives (**95-97**) were first reported in the aerial parts of *D. moldavica* [97,105] and *D. palmatum* (**97**) [103], while malonylated derivatives (**98-100**) were identified in *D. foetidum*, *D. moldavica*, and *D. palmatum* [7,101,103]. The acacetin-7-*O*-*β*-D-glucuronide (**101**) was isolated from dried aerial parts of *D. foetidum*, *D. moldavica*, and *D. ruyschiana* [7,26,105], while its malonyl and acyl derivatives (**102,103**) were detected in *D. foetidum* (**102,103**) and *D. moldavica* (**103**) [7,110].

Among the diglycosides, acacetin-7-*O*-rutinoside (linarin) (**104**) has been detected in the aerial parts of *D. foetidum*, *D. heterophyllum*, *D. palmatum*, and *D. peregrinum* [7,53,94,111], *D. palmatum* with *D. peregrinum* contains its diacetyl derivatives: peregrinumin A (**105**), B (**106**), and C (**107**) [112] (Table S1). Additionally, acacetin-7-*O*-neohesperidoside (**108**) has been identified in *D. moldavica* [105] (Figure 5).

The 7-*O*-substituted acacetin tetraglycosides: acacetin-7-*O*-*β*-D-glucosyl-(1→2)-*β*-D-glucosyl-(1→2)-[*α*-L-rhamnosyl-(1→6)]-*β*-D-glucoside (**109**) and their two acetyl derivatives (**110**,**111**) were identified by Selenge et al. [42] in aerial parts of *D. ruyschiana*, while 8-*C*-glucoside derivatives of acacetin (**112**–**114**) were isolated from aerial parts of *D. palmatum* [26] (Figure 5).

Another monomethoxylated flavone observed in the *Dracocephalum* species is diosmetin (5,7,3′-trihydroxy-4′-methoxyflavone) and its glycosides (**115**) detected in *D. heterophyllum*, *D. moldavica*, *D. palmatum*, and *D. peregrinum* [26,92,94,105] (Figure 6) (Table S1). Diosmetin-7-*O*-glucoside (**116**) has been identified in the aerial parts of *D. heterophyllum*, *D. ruyschiana*, and *D. tanguticum* [26,50,110], while diosmetin-7-*O*-*β*-glucuronide (**117**) has been reported in *D. foetidum* and *D. moldavica* [7,105]. Several diglucosides of diosmetin have also been detected, including diosmetin-7-*O*-rutinoside (**118**) in *D. heterophyllum* [111], diosmetin-7-*O*-neohesperidoside (**119**) in *D. moldavica* [105], and diosmetin-7-*O*-*β*-D-glucose-(1→6)-*β*-D-glucoside (**120**), diosmetin-7-*O*-*α*-L-arabinose-(1→6)-*β*-D-glucoside (**121**), and diosmetin-7-*O*-*β*-D-xylose-(1→6)-*β*-D-glucoside (**122**) in *D. tanguticum* [50]. Moreover, diosmetin tetraglucosides (**123,124**) have been isolated from the aerial parts of *D. ruyschiana* [42].

Other monomethoxylated flavones identified in *Dracocephalum* include dracocephaloside A (7-*O*-(3″-acetyl)-glucoside of 5,8-dihydroxy-4′-methoxyflavone) (**125**) (Figure 7) and geraniol-7-*O*-*β*-D-glucuronide (5,3′-dixydroxy-4′-methoxyflavone-7-glucuronide) (**126**) found in *D. moldavica* [101], genkwanin (5,4′-dihydroxy-7-methoxyflavone) (**127**) found in *D. kotschyi*, *D. multicaule*, and *D. palmatum* [53,96,100], and chrysoeriol (5,7,4′-trihydroxy-3′-methoxyflavone) (**128**) in *D. moldavica* and *D. palmatum* [53,113]. The 5,6,3′,4′-tetrahydroxy-7-methoxyflavone (pedalitin) (**129**), its 3′-*O*-glucoside (**130**), 6-*O*-glucoside (pedaliin) (**131**), pedalin-6′′-acetate (**132**), 5,6,4′-trihydroxy-7-methoxyflavone (ladanetin, scutellarein 7-methyl) (**133**), and its 6-*O*-*β*-D-glucoside (**134)** and 6-*O*-*β*-(6″-*O*-acetyl)glucoside (**135**) (Figure 7) were isolated from *D. tanguticum* [12,102], while 5,6-dihydroxy-7-methoxyflavone (negletein) (**136**) was found in the aerial parts of *D. palmatum* [26].

The dimethoxylated flavones: 5,4′-dihydroxy-6,7-dimethoxyflavone (scrophulein, cirsimaritin) (**137**), and its 4′-*O*-*β*-D-glucoside (**138**), 4′-*O*-glucoside of 5,3′,4′-trihydroxy-6,7-dimethoxyflavone (cirsiliol) (**139**), 5,7,4′-trihydroxy-3′,5′-dimethoxyflavone (**140**), 5,3′,4′-trihydroxy-6,7-dimethoxyflavone (**141**), 5,8,4′-trihydroxy-6,7-dimethoxyflavone (isotymusine) (**142**), and 5,7-dihydroxy-6,4′-trimethoxyflavone (pectolaringenin) (**143**) were detected in *D. multicaule* (**137**), *D. moldavica* (**137**), *D. kotschyi* (**137**), *D. palmatum* (**142**), *D. polychaetum* (**137**), *D. subcapitatum* (**137**), and *D. tanguticum* (**138-141**,**143**) [11,53,95,100,105,108,114,115].

Trimethoxylated flavones: xantomicrol (5,4′-dihydroxy-6,7,8-trimethoxyflavone) (**144**), navadesin (5,7-dihydroxy-6,8,4′-trimethoxyflavone) (**145**), salvigenin (5-hydroxy-6,7,4′-trimethoxyflavone) (**146**), 8-hydroxy-salvigenin (5,8-dihydroxy-6,7,4′-trimethoxyflavone) (**147**), and circlineol (5,4′-dihydroxy-7,8,3′-trimethoxyflavone) (**148**) have been identified in aboveground parts of *D. kotschyi* (**144,148**), *D. moldavica* (**144,146,147***)*, *D. multicaule* (**144,148**), *D. palmatum* (**145,146**), *D. peregrinum* (**146**), *D. polychaetum* (**144,148**), and *D. subcapitatum* (**144,148**) [26,53,62,96,100,104,105,109,114,116] (Figure 8) (Table S1).

Among the tetramethoxylated flavones, santaflavone (5-demethylsinensetin, 5-hydroxy-6,7,3′,4′-tetramethoxyflavone,) (**149**) was identified in *D. moldavica*, *D. kotschyi*, *D. multicaule*, *D. peregrinum*, *D. polychaetum*, and *D. subcapitatum* [104,109,114]. Gardenin B (5-hydroxy-6,7,8,4′-tetramethoxyflavone) (**150**) was detected in *D. moldavica* [110] and *D. multicaule* [100]. Gardenin A (5-hydroxy-6,7,8,3′,4′,5′-hexamethoxyflavone) (**151**) was identified in *D. moldavica* [104] (Figure 9) (Table S1).

The genus *Dracocephalum* also produces flavones that are 3-*O*-methoxylated derivatives of flavonols, such as 5,7,4′-trihydroxy-3-methoxyflavone (isokaempferide) (**152**), detected in *D. kotschyi*, *D. multicaule*, and *D. subcapitatum* [62,96,100], 5,4′-dihydroxy-3,7-dimethoxyflavone (kumatakenin) (**153**) detected in *D. multicaule*, 5,7,4′-trihydroxy-3,3′-dimethoxyflavone (**154**) detected in *D. kotschyi*, *D. multicaule*, *D. polychaetum*, and *D. subcapitaum*, 5,3′,4′-trihydroxy-3,7-dimethoxyflavone (**155**) detected in *D. peregrinum*, and 5,7-dihydroxy-3,4′-dimethoxyflavone (ermanin) (**156**) detected in *D. kotschyi*, *D. multicaule*, *D. polychaetum*, and *D. subcapitaum* [94,100,114] (Table S1) (Figure 10). More compounds with 3-*O*-methoxyled group and a larger number of methoxy groups were penduletin (5,4′-dihydroxy-3,6,7-trimethoxyflavone) (**157**) reported in *D. kotschyi and D. multicaule* [96,100], chrysosplentin (5,4′-dihydroxy-3,6,7,3′-tetramethoxyflavone) (**158**) found in *D. heterophyllum* [111], calicopterin (5,4′-dihydroxy-3,6,7,8-tetramethoxyflavone) (**159**) recorded in *D. kotschyi*, *D. multicaule*, *D. polychaetum*, and *D. subcapitatum* [62,96,100,114], and 4′-methylcalicopterin (5-hydroxy-3,6,7,8,4′-pentamethoxyflavone) (**160)** identified in *D. multicaule* [100] (Table S1) (Figure 10).

#### 5.2.2. Flavonols and Their Sugar Derivatives

The genus *Dracocephalum* has been found to exhibit flavonol compounds, such as 3-hydroxyflavone which was detected in *D. moldavica* (**161**) [104] and gossypetin (5,7,8,3′,4′-pentahydroxyflavone-3-*O*-rhamnosyl-(1-6)-glucoside) (**162**) was identified in aerial parts of *D. heterophyllum* [12] (Table S1) (Figure 11).

The aglycones kaempferol (3,5,7,4′-tetrahydroxyflavone) (**163**) and quercetin (3,5,7,3′,4′-pentahydroxyflavone) (**164**) were also detected in the aerial parts of *D. heterophyllum* (**163,164**), *D. kotschyi* (**164**), *D. moldavica* (**163,164**), *D. palmatum* (**163**), *D. peregrinum* (**163,164**), *D. polychaetum* (**164**), and *D. ruyschiana* (**163**) [10,12,13,26,94,105,111] (Table S1) (Figure 11).

Kaempferol glycosides substituted at positions 3 and 7, such as kaempferol-7-*O*-glucoside (**165**), astragalin (kaempferol-3-O-glucoside) (**166**), kaempferol-3-*O*-rhamnoside (**167**), kaempferol-3-*O*-glucuronide (**168**), and kaempferol-3-*O*-rutinoside (**169**), have been reported in *D. moldavica* (**165,166**), *D. palmatum* (**166,169**), *D. peregrinum* (**166,167)**, and *D. ruyschiana* (**166,168**) [26,42,94,97]. Moreover, several kaempferol derivatives, including 2”-coumarylglucoside astragalin (**170**), kaempferol-3-*O*-*β*-D-(6”-*O*-p-coumaroyl)-galactoside (**171**), and 3-*O*-rhamnoside of kaempferide (3,5,7-trihydroxy-4′-methoxyflavone) (**172**), have been identified in *D. moldavica* (**170,171**) and *D. peregrinum* (**172**) [12,94] (Table S1) (Figure 11).

The quercetin glycosides, including quercetin-7-*O*-*β*-D-glucoside (**173**), quercetin-3-*O*-*β*-D-glucoside (**174**), quercetin-3-O-β-D-rhamnoside (quercitrin) (**175**), quercetin-3-*O*-galactoside (**176**), quercetin-3-*O*-rutinoside (**177**), and quercetin-3-*O*-*β*-D-glucuronide (**169**), were identified in *D. moldavica* (**174-176**), *D. peregrinum* (**173-175**), *D. ruyschiana* (**174,178**), and *D. tanguticum* (**177**) [42,93,94,97,105,117]. The 3′-methoxylated derivative of quercetin, isorhamnetin (**179**), was found in D. *moldavica* [12] (Table S1) (Figure 11).

#### 5.2.3. Flavanone Derivatives

Among the flavanones, eriodictyol (5,7,3′,4′-tetrahydroxyflavan-4-one) (**180**) and its derivatives, such as eriodictyol-7-*O*-*β*-D-glucoside (**181**), eriodictyol-7-*O*-(4′′-*O*-malonyl) -*β*-D-glucoside (**182**), eriodictyol-7-O-rutinoside (**183**), and eriodictyol-7-*O*-rutinoside (6′′-*O*-malonyl)-*β*-D-glucoside (**184**), have been reported in *D. palmatum* [25,53,103]. Additionally, eriodictyol was detected in *D. peregrinum*, *D. rupestre*, and *D. tanguticum* [55,93,102], and its glucoside in *D. rupestre*, *D. ruyschiana*, and *D. tanguticum* [26,55,102] (Table S1) (Figure 12).

The compounds naringenin (5,7,4′-trihydroxyflavan-4-one) (**185**) and prunin (naringenin-7-*O*-glucoside) (**186**) were detected in *D. kotschyi* (**185**), *D. moldavica* (**185**), *D. palmatum* (**185,186**), *D. polychaetum* (**185**), *D. rupestre* (**185,186**), *D. ruyschiana* **(186**), and *D. tanguticum* (**185,186**) [13,25,26,55,98,102,118]. Additionally, narirutin (7-*α*-L- rhamnopyranosyl-(1→6)-*β*-D-glucosylo-5,7,4′-trihydroxyflavan-4-one) (**187**) was identified in the aerial parts of *D. tanguticum* [99], while 5,7,3′,5′ -tetrahydroxyflavan-4-one (**188**) and its 7-*O*-*β*-D-glucoside (**189**) were detected in *D. tanguticum* [102].

#### 5.2.4. Flavanonol Derivatives

Dihydroquercetin (3,5,7,3′,4′,-pentahydroxyflavan-4-one) (**190**), dihydrokaempferol (3,5,7,4′-tetrahydroxyflavan-4-one) (**191**), and ampelopsin (3,5,7,3′,4′,5′-hexahydroxyflavan-4-one) (**192**) were identified in *D. palmatum* [26] (Figure 13). Another flavanonol compound, fustin (3,7,3′,4′-tetrahydroxyflavan-4-one) (**193**), was detected in *D. ruyschiana* [26] (Table S1).

#### 5.2.5. Flavanol Derivative

Two flavan-3-ols have been detected in the *Dracocephalum* species: catechin (**194**) in *D. ruyschiana*, *D. moldavica*, *D. kotschyi*, and *D. polychaetum*, and gallocatechin (**195**) in *D. palmatum* [13,26,92] (Figure 14) (Table S1). These molecules serve as precursors for the synthesis of polymers such as proanthocyanidins and anthocyanidins.

### 5.3. Anthocyanins

**Figure 15:**
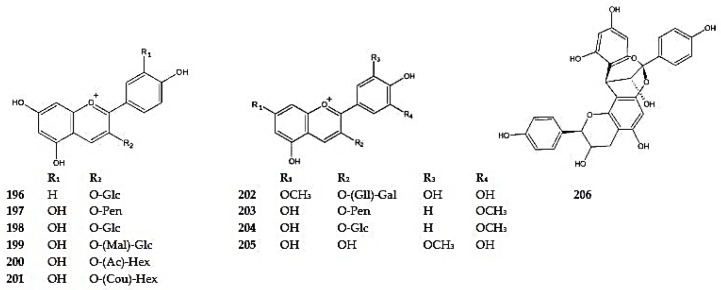
Anthocyanidins detected in *Dracocephalum* species (Ac—acetyl, Cou—coumaroyl, Gal—galactoside, Glc—glucoside, Gll—galloyl, Hex—hexoside, Mal—malonyl, Pen—pentoside).

Various anthocyanins have been identified in the genus *Dracocephalum*. The following have been detected in *D. palmatum*: pelargonidin 3-*O*-glucoside (callistephin) (**196**), cyanidin *O*-pentoside (**197**), cyanidin 3-*O*-glucoside (**198**), and its acetylated, malonylated, and coumaroylated derivatives cyanidin 3-*O*-(6″-malonyl)glucoside (**199**), cyanidin 3-*O*-(acetyl)hexoside (**200**), cyanidin 3-*O*-(coumaroyl)hexoside (**201**), 7-*O*-methyl-delphinidin 3-*O*-(2″galloyl)-galactoside (**202**), peonidin-*O*-pentoside (**203**), peonidin 3-*O*-glucoside (**204**), and petunidin (**205**) [26]. In addition, petunidin and pelargonidin 3-*O*-glucoside have also been reported in *D. ruyschiana* (Figure 15) (Table S1). Furthermore, the presence of a condensed tannin, procyanidin A-type dimer (epicatechin-catechin dimer) (**206**), has been identified in *D. ruyschiana* [26].

### 5.4. Lignans

**Figure 16:**
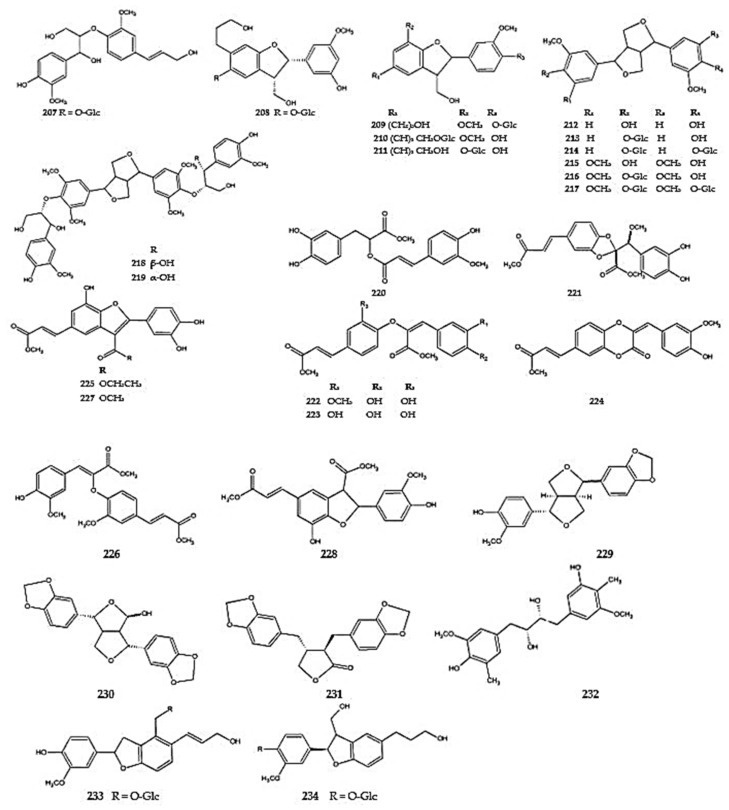
Lignans detected in *Dracocephalum* species (Glc—glucoside).

The lignans have been found primarily in *D. moldavica*, and they might be considered characteristic of the species. The following compounds have been isolated from the aerial parts of *D. moldavica*: alaschanisoside A (**207**), akequintoside A (**208**), (7,8)-dihydrodehydrodiconiferyl alcohol 4-*O*-*β*-D-glucoside (**209**), (7,8)- dehydrodiconiferyl alcohol 9′-*O*-*β*-D-glucoside (**210**), (7,8)-3′-demethyl-dehydrodiconiferyl alcohol 3′-*O*-*β*-D-glucoside (**211**), (+)-pinoresinol (**212**), syringaresinol (**215**), hedyotisol A (**218**) and B (**219**), oresbiusin B (**220**), dracomolphins A-E (**221-225**), methyl-3-(4-hydroxy-3-methoxyphenyl)-2-{2-methoxy-4[(-3-methoxy-3-oxoprop-1-enyl]phenoxy}-prop-2-enoate (**226**), 3-benzofurancarboxylic acid-2-(3,4-dihydroxyphenyl)-7-hydroxy-5-(3-methoxy-3-oxo-1-propenyl)-methyl ester (**227**), and 3-[2-(3,4-dihydroxyhenyl)-7-hydroxy-3-methoxycarbonyl-2,3-dihydro-1-benzofuran-5-yl]prop-2-enoate (**228**) [12,29,119]. Two other lignans, viz. (+)-piperitol (**229**) and (9+)-9*α*-hydroxysesamin (**230**), were described in the same species by Jöhrer et al. [116] (Figure 16) (Table S1).

The lignans, pinoresinol mono- and diglucosides (**213,214**), and syringaresinol glucosides (**216,218**) were detected in *D. heterophyllum* (**213,214,216**), *D. moldavica* (**216,217**), and *D. tanguticum* (**216**) [12,29], hinokinin was identified (**231**) in *D. ruyschiana*, and dimethyl-secoisolariciresinol (2,3-dimethyl-2,3-di(4-hydroxy-3-methoxybenzyl)-1,4-butanediol) (**232**) was identified in *D. ruyschiana* and *D. palmatum* [26]. The little-known lignans dehydropine-9-*β*-glucoside (**233)** and 2,3-dihydro-7-hydroxy-2-(4′-hydroxy-3′methoxophenyl)-3-hydroxymethyl-5-benzofuranpropranol-4′-*O*-*β*-D-glucoside (**234**) were identified in *D. heterophyllum* (**233**) and *D. tanguticum* (**234**) [83,120] (Figure 16) (Table S1).

### 5.5. Other Compounds

**Figure 17:**
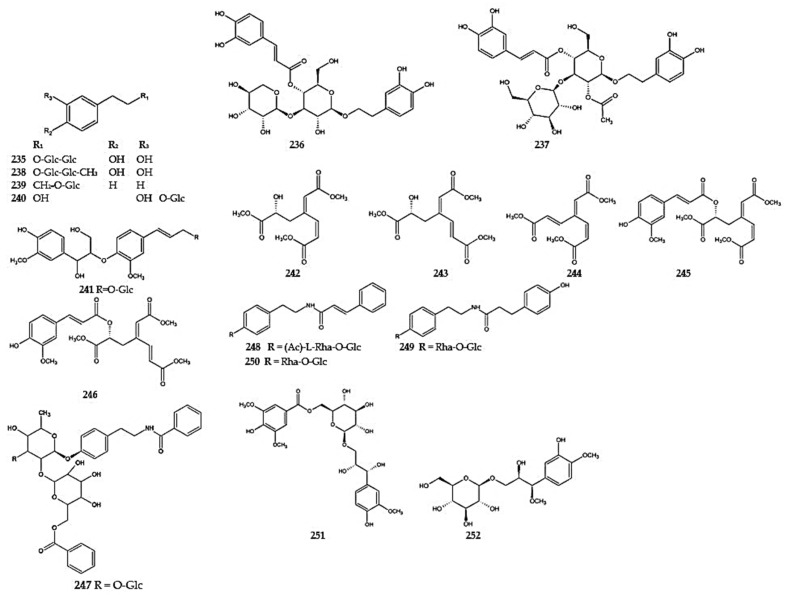
Phenylethanoids, seco-phenylpropanoids, and other phenylpropanoids detected in *Dracocephalum* species (Ac—acetyl, Glc—glucoside, Rha—rhamnoside).

Several phenylethanoid compounds have been found in the genus *Dracocephalum*. Ferruginoside B (**235**), verbascoside (acteoside), and (**236**) 2′-*O*-acetyl plantamajoside (**237**) were isolated from *D. heterophyllum* [87]. The presence of the latter two compounds in this species, together with decaffeoylverbascoside (**238**), was confirmed by subsequent studies [88]. Compounds from this group were also detected in *D. tanguticum*, including 3-phenylethyl-*β*-D-glucoside (**239**) [115], followed by osmanthuside F (**240**) and hyuganoside IIIa (**241**) [83](Table S1) (Figure 17).

Five 3,4-seco-phenylpropanoids with an unusual aromatic ring cleaved skeleton named dracomolphesin A–E (**242**–**246**) were isolated from aerial parts of *D. moldavica* [121]. Four dratanguticumide A–D (**247**–**250**) as phenylacetamide glycosides and two dratanguticumide G (**251**) and H (**252**) as phenylpropanoid glycosides were obtained from *D. tanguticum* [83,84] (Figure 17) (Table S1).

**Figure 18:**
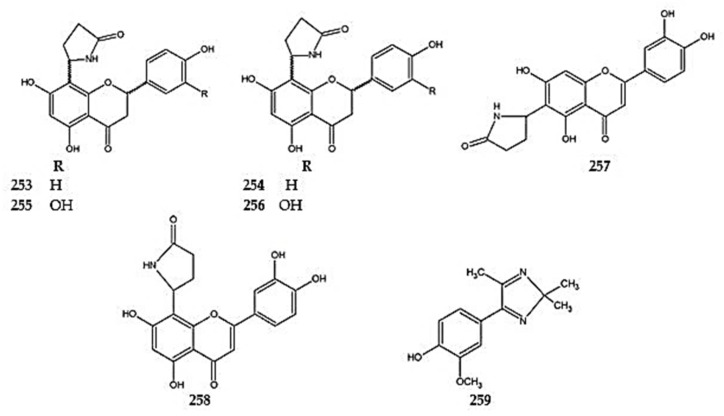
Flavonoid alkaloids detected in *Dracocephalum* species.

Phytochemical studies on the aerial parts of *D. rupestre* led to the isolation of dracocephins A–D, i.e., flavonoid alkaloids (**253**–**256**). Dracocephins are conjugates of naringenin (A and B) or eriodictyol (C and D) with pyrrolidin-2-one. They were found to exist as mixtures of two diastereoisomeric pairs of enantiomers with two chiral centers, C-2 and C″-5 [122]. Two additional flavonoid alkaloids, drahebephins A (**257**) and B (**258**), along with an imidazole alkaloid containing a phenolic substituent, drahebenine C (**259**), were isolated from the aerial parts of *D. heterophyllum* [92] (Figure 18) (Table S1).

**Figure 19:**
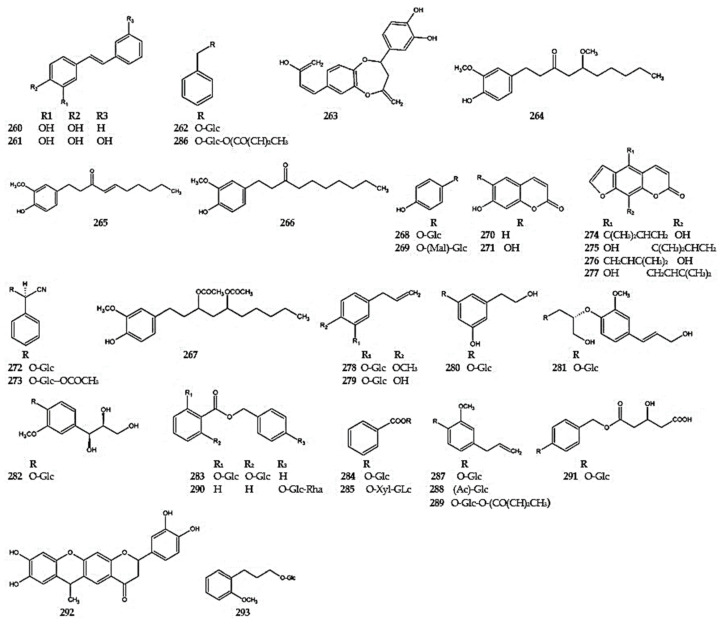
Others phenolic compounds detected in *Dracocephalum* species (Glc—glucoside, Mal—malonyl, Rha—rhanoside, Xyl—xyloside).

In addition, other phenolic compounds have been identified in several *Dracocephalum* species. Stilbenes such as pinosylvin (**260**) and resveratrol (**261**) [26] and 5-benzyl alcohol glucoside (**262**) [42] were detected in *D. ruyschiana* (Figure 19).

Sibiricin A (**263**) and four gingerols—5-methoxy-6-gingerol (**264**), 6-shogaol (**265**), 6-paradol (**266**), and diacetoxy-6-gingeridol (**267**)—were isolated from the extract of *D. heterophyllum* [87,123]. Arbutin (**268**) and *O*-malonylarbutin (**269**) [53], as well as the coumarins umbelliferone (**270**) and aesculetin (**271**) [25] were reported in *D. palmatum*. Moreover, compound *O*-malonylarbutin was detected in *D. moldavica* [30]. Meanwhile, cyanogenic glucosides, prunasin (**272**), and peregrinumcin A (**273**) were isolated from *D. peregrinum* [112]. Additionally, four furanocoumarins were identified in *D. heterophyllum* (**274**–**277**) [124].

In the aerial parts of *D. moldavica*, citrusin C (**278**), 4-allyl-1-[(*β*-D-glucosyl)oxy]-2-hydroxybenzene (**279**), 3,5-dihydroxyphenethyl alcohol 3-*O*-*β*-D-glucoside (**280**), and 3-hydroxy-2-[4-(3-hydroxy-1-propen-1-yl)-2-methoxyphenoxy]propyl-*β*-D-glucoside (**281**) were identified [29]. Citrusin C was also obtained from *D. ruyschiana* [42].

Moreover, in *D. tanguticum* is the presence of guaiacyl glycerol-3′-*O*-*β*-D-glucopyranoside (**282**), benzyl benzoate-2,6-di-*O*-*β*-D-glucoside (**283**), benzyl-*β*-D-glucoside (**284**), benzyl-*O*-*β*-D-xylosyl-(1→6)-*β*-D-glucoside (**285**), benzyl-6-[(2*E*)-2-butenoate]-*β*-D-glucoside (**286**), 2-methoxy-4-(2-propen-1-yl)phenyl-*β*-D-glucoside (**287**), 2-methoxy-4-(2-propen-1-yl)phenyl-6-acetate-*β*-D-glucoside (**288**), and 2-methoxy-4-(2-propen-1-yl)phenyl-6-[(2*E*)-2-butenoate]-*β*-D-glucoside (**289**) [83,108]. Additionally, in *D. ruyschina*, [(benzoxy)methyl]phenyl-4-*O*-*α*-L-rhamnosyl(1-6)-*O*-*β*-glucoside (**290**) and [(3-hydroxy-3-methoxyglutaryl)methyl]phenyl-4-*O*-*β*-D-glucoside (**291**) were identified [42]. In *D. rupestre* dracorupesins A (**292**) has also been reported, and in *D. tanguticum* taettoside E (**293**), has been identified [115,125] (Figure 19).

## 6. Pharmacological Properties of *Dracocephalum*

Recent studies performed with the use of more modern methods have provided a more accurate understanding of the use of several *Dracocephalum* species in traditional medicine. Table 3 presents data on the main biological activities of *Dracocephalum* resulting from the presence of phenolic compounds.

**Table 3:** Biological activity of selected *Dracocephalum* species.

Biological Activity	Species	Plant Material	Extracts/Fractions or Compounds	Assays Model	Results	References
Antioxidant	*D. foetidum*	aerial parts	acetone, different fractions, phenolic compounds	DPPH	EC_50_ = 9.2–66.1 µg/mL (acetone extract and fractions), EC_50_ = 6.8–223 µM (compounds)	[7]
	*D. heterophyllum*	aerial parts	rosmarinic acid, fenylethanoids	DPPH	EC_50_ = 19.37–26.63 µM	[88]
		aerial parts	furanocoumarins	DPPH, cellular antioxidant activity assays	EC_50_ = 115.36–284.33 µM, expression of Nrf_2_↓	[124]
		aerial parts	gingerols	DPPH, cellular antioxidant activity assays	EC_50_ = 2.95–18.68 µM, expression of antioxidant enzymes↓	[123]
		aerial parts	different fractions	^(1)^ DPPH, ^(2)^ABTS, ^(3)^ FRAP, ^(4)^ Fe^3+^ chelation, ^(5)^ LPO	inhibition: ^(1)^ 9.1–88.8% (50 µg/mL), ^(2)^ 8.0–75.2% (50 µg/mL), ^(3)^ 0.05–1.6 mM/L FeSO_4_, ^(4)^ 21.8–72.2% (0.5 mg/mL), ^(5)^ 67.5–98.7% (50 µg/mL)	[126]
		flowers, leaves, roots	80% methanol	DPPH	EC_50_ = 37 μg/mL	[4]
	*D. kotschyi*	leaves, flowers	different extracts	^(1)^ DPPH, ^(2)^ FRAP	^(1)^ EC_50_ = 1.02–2.84 mg/mL (leaves), ^(1)^ EC_50_ = 3.17–5.4 mg/mL (flowers), ^(2)^ 0.19–0.64 mM Fe(II)/g DW (leaves), ^(2)^ 0.12–0.4 mM Fe(II)/g DW (flowers)	[14]
		aerial parts	ethanol	DPPH	EC_50_ = 50 µg/mL	[13]
		leaves, flowers	methanol	DPPH	EC_50_ = 15.84–60.72 µg/mL (flowers), EC_50_ = 46.06–105.52 µg/mL (leaves)	[127]
		aerial parts	different extracts	^(1)^ DPPH, ^(2)^ FRAP	^(1)^ EC_50_ = 23.2–1584.0 µg/mL, ^(2)^ 6.65–80.7 µM mg FSE/g extract	[128]
		aerial parts	different extracts	^(1)^ DPPH, ^(2)^ ABTS, ^(3)^ FRAP	^(1)^ EC_50_ = 9.55–46.2 μg/mL, ^(2)^ EC_50_ = 7.47–51.14 µM Trolox/g, ^(3)^ EC_50_ = 6.87–106.35 µM Trolox/g	[64]
		aerial parts	different extracts	^(1)^ DPPH, ^(2)^ FRAP	^(1)^ EC_50_ = 4.85–252.48 μg/mL, ^(2)^ 2.5–32.357 mM Fe^2+^/g DW	[129]
		aerial parts	different extracts	DPPH	EC_50_ = 0.0899–2.3 μg/mL	[130]
		leaves of various populations	diethyl ether	FRAP	38.71–1681.41 µM Fe^2+^/100 g DW	[11]
	*D. moldavica*	aerial parts of three different cultivars	70% ethanol	^(1)^ DPPH, ^(2)^ FRAP	^(1)^ EC_50_ = 35.542–40.901 μg/mL, ^(2)^ 293.194–330.165 μM Trolox mg DW	[98]
		aerial parts	different extracts	^(1)^ DPPH, ^(2)^ CLB	^(1)^ EC_50_ = 35.542–40.901 μg/mL, ^(2)^ inhibition: 59–94% at 150 μg/mL	[93]
		aerial parts	ethyl acetate	^(1)^ DPPH, ^(2)^ ABTS, ^(3)^ O_2_, ^(4)^ OH, ^(5)^ SOD	activity values about: ^(1)^ 5–75%, ^(2)^ 8–95% (extract concentration unknown), ^(3)^ 30–95 U/mL, ^(4)^ 25–100 U/mL, ^(5)^ 50 U/mg proteins	[131]
		leaves	alcoholic extracts	^(1)^ DPPH, ^(2)^ FRAP, ^(3)^ O_2_^∙ (4)^ NO, ^(5)^ H_2_O_2_	^(1)^ EC_50_ = 25.96– 87.77 μg/mL, ^(2)^ 101.40–185.437 μmol/100g, ^(3)^ 44.43–57.14% (at 20 mg/mL), ^(4)^ 37.60–87.90% (at 20 mg/mL), ^(5)^ 4.48–7.93% (at 2 mg/mL)	[33]
		aerial parts	hydromethanolic	^(1)^ DPPH, ^(2)^ABTS, ^(3)^ O_2_, ^(4)^ OH, ^(5)^ Fe^3+^ chelation, ^(6)^ reducing power assay	^(1)^ EC_50_ = 23.1 µg/mL, ^(2)^ EC_50_ = 8.0 µg/mL, ^(3)^ EC_50_ = 445.5 µg/mL, ^(4)^ EC_50_ = 269.3 µg/mL, ^(5)^ EC_50_ = 35.70 µg/mL, ^(6)^ EC_50_ = 17.07 µg/mL	[68]
		aerial parts	total flavonoids	^(1)^ DPPH, ^(2)^ O_2,_ ^(3)^ OH	inhibition: ^(1)^ 75%, ^(2)^ 85%, ^(3)^ 85% (at 100 mg/mL)	[132]
		aerial parts	acacetin-7-*O*-(4″-acetyl)-glucoside	DPPH	EC_50_ = 98 µM.	[97]
		aerial parts	different extracts	^(1)^ DPPH, ^(2)^ ABTS, ^(3)^ Fe^3+^ reduction, ^(4)^ CLB	^(1)^ 47.4–85.9% (1 mg/mL), ^(2)^ 0.5–1.04 mM Trolox, ^(3)^ 137.07–311.07 µM/g, ^(4)^ inhibition: 1.9–19.2% (at 1 mg/mL)	[10]
		aerial parts	different extracts	DPPH	IC_50_ = 24.5 μg/mL	[133]
	*D. multicaule*	aerial parts	different extracts	^(1)^ DPPH, ^(2)^ FRAP	^(1)^ EC_50_ = 25.0–707 µg/mL, ^(2)^ 9.93–229.92 µM mg FSE/g extract	[128]
		flowering tops	methanol	DPPH	EC_50_ = 156.5 µg/mL	[134]
		plant	methanol	^(1)^ DPPH, ^(2)^ ABTS, ^(3)^ FRAP	^(1)^ EC_50_ = 1.41 mg/mL, ^(2)^ IC_50_ = 2.11 mg/mL (in 6 min), ^(3)^ IC_50_ = 1.41 µg/mL	[135]
	*D. palmatum*	aerial parts	luteolin-7-O-glucoside	^(1)^ DPPH, ^(2)^ ABTS, ^(3)^ Br, ^(4)^ O_2_, ^(5)^ NO, ^(6)^ H_2_O_2_, ^(7)^ Fe^2+^ chelation	^(1)^EC_50_ = 16.97 µM/mL, ^(2)^ EC_50_ = 9.86 µM/mL, ^(3)^ 1000 (mg-eq) luteolin-7-*O*-glucoside/g, ^(4)^ EC_50_ = 14.92 µM/mL, ^(5)^ EC_50_ > 100 µM/mL, ^(6)^ 0.53 mM/g ^(7)^ 106.12 mM Fe^2+^/g	[53]
		aerial parts	ethanol	^(1)^ DPPH, ^(2)^ ABTS, ^(3)^ Br, ^(4)^ O_2_, ^(5)^ CLB, ^(6)^ NO, ^(7)^ H_2_O_2_, ^(8)^ FRAP, ^(9)^ Fe^2+^ chelation, ^(10)^ EMSA	^(1)^ EC_50_ = 12.7–18.6 µM/mL, ^(2)^ EC_50_ = 6.4–10.8 µM/mL, ^(3)^ 247.7–389.7 mg cynaroside/g, ^(4)^ EC_50_ = 19.4–28.6 µM/mL, ^(5)^ EC_50_ = 1.6–3.4 µM/mL, ^(6)^ EC_50_ = 29.3–41.8 µM/mL, ^(7)^ 1.2–2.03 mM/g, ^(8)^ 12.2–22.3 mM Fe^2+^/g, ^(9)^ EC_50_ = 30.9–48.1 µM/mL, ^(10)^ EC_50_ = 14.1–51.6 µM/mL	[25]
	*D. polychaetum*	aerial parts	ethanol	DPPH	EC_50_ = 175 µg/mL	[13]
		aerial parts	methanol	DPPH	inhibition: 68–85% at 50–1000 ppm of extract	[136]
		aerial parts	different extracts	^(1)^ DPPH, ^(2)^ FRAP	^(1)^ EC_50_ = 50.5–5346.1 µg/mL, ^(2)^ 4.3–16.43 µM mg FSE/g extract	[128]
		aerial parts	80% methanol	^(1)^ DPPH, ^(2)^ FRAP	^(1)^ EC_50_ = 5.6 mg/mL, ^(2)^ EC_50_ = 0.156 mg/mL	[60]
		aerial parts	different extracts	DPPH	EC_50_ = 8.07–24.04 mg/mL	[79]
	*D. rupestre*	plant	ethyl acetate	^(1)^ DPPH, ^(2)^ ABTS, ^(3)^ OH, ^(4)^ FRAP, antioxidant enzyme assays	^(1)^ EC_50_ = 50.01 µg/mL, ^(2)^ EC_50_ = 43.62 µg/mL, ^(3)^ EC_50_ = 28.59 µg/mL, ^(4)^ about 50 g Trolox/100 g DW	[57]
		plant	eriodictyol-7-*O*-glucoside	cisplatin-induced oxidative stress in HRMC	activation of Nrf2 pathway↑, glutathione level↑, cell survival rate↑	[80]
	*D. ruyschiana*	aerial parts	phenolic compounds	DPPH	EC_50_ = 28.9–328 µM	[42]
	*D. tanguticum*	plant	isolated phenolic compounds	^(1)^ DPPH, ^(2)^ ABTS, ^(3)^ Fe^2+^ reduction	^(1)^ EC_50_ = 6.12–113 µM, ^(2)^ EC_50_ = 1.15–400 µM, ^(3)^ EC_50_ = 0.06–515 µM	[102]
Anti- inflammatory	*D. foetidum*	aerial parts	acetone, fractions, isolated compounds	hyaluronidase activity	hyaluronidase activity↓, EC_50_ = 0.27–0.35 mg/mL (extract and its fractions), EC_50_ = 0.19–0.79 mM (compounds)	[7]
	*D. heterophyllum*	aerial parts	ethyl acetate	animal model (mice)	synovial inflammation↓, joint swelling↓, cartilage and bone destruction↓, anti-CII IgG↓, TNF-α↓, IL-6↓, IL-1β↓	[137]
		plant	ethyl acetate	animal model (mice)	ALT↓, AST↓, TBA↓ lobular inflammation↓, col1α1↓, α-SMA↓, MCP-1↓, CD68↓	[46]
		plant	ethyl acetate	animal model (mice)	CD4^+^ T cells↓, IFN-γ↓, IL-17A↓, IL-2↓, IL-6↓, the inhibitory phosphorylation (Tyr15) of Cdc2↑, expression Wee1 kinase↑, retinal edema↓, structural distortion↓, inflammatory cell infiltration↓	[120]
		aerial parts	different fractions	animal model (mice)	mortality↓, ALT↓, AST↓, cell apoptosis and necrosis↓	[126]
		plant	ethanol	animal model (mice)	TNF-*α*↓, IFN-*γ*↓, ALT↓, AST↓, hepatic damage and inflammatory infiltrates↓, number of apoptotic cells↓, D11b+ Gr1+ MDSC in liver↑, macrophage activation↓	[138]
	*D. kotschyi*	leafy shoots	70% alcohol, water	animal model (rats)	lung tissues necrosis↓, inflammation↓, fibrosis↓, hydroxyproline↓, MDA↑	[139]
		aerial parts	70% ethanol	animal model (rats)	MPO activity↓, ulcer area and index↓, colonic weight/length ratio↓, total colitis index↓	[140]
		aerial parts	methanol, calycopterin	PBMNs	lymphocyte proliferation↓	[66]
		plant	methanol	mouse macrophages	NO↓, IL-1β↓	[141]
	*D. moldavica*	plant	ethyl acetate	animal model (rats)	body weight loss↓, DSS-induced tissue morphological changes↓. members of the TLR4/NF-κB signaling pathway↓, NF-κB↓, IL-17↓, TNF↓, Toll-like receptor (TLR) signaling pathways↓, *Lactobacillus*↑, *Romboutsia*↓	[142]
		-	total flavonoid fraction	animal model (mice)	lung tissue fibrosis ↓, IL-6↓, TNF-α↓, Col-I↓, FN1↓, α-SMA↓, Shh↓, Ptch1↓, SMO↓, Gli1↓, SUFU↑	[143]
		plant	50% ethanol	animal model (rats)	CDS↓, myeloperoxidase↓, nitric oxide↓, MDA↓, total protein↑, TNFα↓, IFNɣ, IL-6↓, IL-4↑,	[144]
		leaves	70% ethanol	RAW 264.7 cells, animal model (mice)	NO↓ PGE2↓, iNOS↓, COX-2↓, IL-6↓, IL-1β↓, the inhibition of the ERK/JNK/NF-κB signaling	[145]
		aerial parts	total flavonoid extract	rat vascular smooth muscle cells	VSMC proliferation and migration↓, PCNA expression↓, expression of ICAM-1 and VCAM-1↓, NF-κB p65 expression↓	[146]
	*D. peregrinum*	plant	peregrinumin A, B, C, peregrinumcin A	RAW 264.7 cells	NO↓, NF-κB↓(peregrinumin B and C)	[112]
		aerial parts	1′-methyl-2′-hydroxyethyl ferulate	RAW 264.7 cells	NO↓	[94]
	*D. rupestre*	aerial parts	60% ethanol (*D. rupestre* + *Berberidis* radix)	animal model (mice with *Salmonella*-induced diarrhea)	IL-6↓, sigA↓, TNF-α↓, mucosal repair markers (PCNA, TGF-β1, and EGFR)↑, beneficial bacterial genera	[147]
		plant	ethyl acetate fraction	animal model (mice)	ALT↓, AST↓, MDA↓, LDH↓, necrotic areas↓	[57]
	*D. tanguticum*	plant	different extracts	animal model (mice), human hepatic LO2 cell	Nrf2↑, NF-κB↓, HO-1↑, NQO-1↑, GSTA1↑, IKB2↑, Keap1↓, ALT↓, AST↓, hepatic lipid indicators (TC↓, TG↓, LDL-C↓,HDL-C↑), lipid peroxidation indicators (ADH↑, MDA↓, SOD↑, CAT↑, and GSH-Px↑), necrosis of hepatocytes↓, lymphocyte infiltration↓	[148]
Neuroprotective and neuromodulatory	*D. kotschyi*	aerial parts	different extracts	^(1)^ anti-tyrosinase assay, ^(2)^ anti-AChE assay, ^(3)^ anti-BChE assay	inhibition (at con. of 1 mg/mL): ^(1)^ 11.7–51%, ^(2)^ 17.7–63%, ^(3)^ 11.1–47%	[128]
	*D. moldavica*	plant	total flavonoid fraction	animal model (mice)	IL-1β, IL-6 and TNF-α↓, SRC/β-acttin↓	[104]
		leaves	ethanol	animal model (mice)	step-through latency in the retention trial↑, latencies in the Morris water maze task↓	[149]
		plant	flavonoid fraction	U87 cells	cell viability↑, apoptotic rate↓, LDH↓, MDA↓, caspase-3↓, caspase-12↓, Bcl-2↓, Bax↓, ROS↓,	[150]
		aerial parts	ethanol	animal model (mice)	immobility time in the FST↓ swim time in FST ↑, immobility time TST↓,	[71]
		plant	total flavonoid fraction	animal model (rats)	GSH/GSSG ratio↑, SOD↑, the GPX↑, CAT↑, MDA↓, carbonyl↓, 8-OHdG↓, TUNEL-positive cells↓, neurological deficit↓,	[151]
		aerial parts	water	animal model (mice)	pentobarbital-induced sleeping time↑, sedation in the HBT↑, spontaneous activity↓, motor coordination impairment↑, the immobility time↑	[30]
	*D. multicaule*	aerial parts	70% ethanol	animal model (rats)	hippocampal neuronal cell density↑, time spent in the target quadrant↑	[59]
		aerial parts	different extracts	^(1)^ anti-tyrosinase assay ^(2)^ anti-AChE assay, ^(3)^ anti-BChE assay	inhibition (at conc. 1 mg/mL) ^(1)^ 19.8–50%, ^(2)^ 23–97%, ^(3)^ 15–46.8%	[128]
		flowering aerial parts	methanol	anti-AChE assay	IC_50_ = 1.06 µg/mL	[134]
	*D. polychaetum*	aerial parts	different extracts	^(1)^ anti-tyrosinase assay, ^(2)^ anti-AChE assay, ^(3)^ anti-BChE assay	inhibition (at conc. 1 mg/L): ^(1)^ 6.5 -39.5%, ^(2)^ 10–48.6%, ^(3)^ 21.7–62.7%	[79]
	*D. ruyschiana*	aerial parts	water	clinical study (patients with mild to moderate depression	the mean score of depression by BDI (Beck Depression Inventory)↓	[152]
	*D. tanguticum*	aerial parts	n-butanol	animal model (rats)	in the IFC and hippocampus: BDNF mRNA↑, NT-3 mRNA↑, Bederson and Longa scoring↓, beam-walking test↓, GPx↑, CAT↑, SOD↑, MDA↓	[51]
Cardioprotective anti- ischemic, anti-hypertension	*D. heterphyllum*	plant	purified 70% methanolic extract containing flavonoids	neonatal rat cardiomyocytes	cell survival rate↑, c-jun↓, BNP↓, ANP↓, *β*-MHC↓, Ca^2+^↓, eNOS↑, NO↑	[45]
	*D. moldavica*	-	extract containing flavonoids	H9c2 cells	LDH↓, SOD↑, MDA↓, ROS↓, proliferation of cell↑, Bax↓, Bcl-2↑, NOX-4↓, p-P38↓, MAPK/P38 MAPK↓, p-Erk1/2/Erk1/2↓, PGC-1α↑	[101]
		aerial parts	ethyl acetate	animal model (rats)	SBP↓, ROS↓, dysregulation of miRNA expression↓, gut microbiota dysbiosis↓	[32]
		-	isolated compounds	animal model (mice)	blood glucose↓, the running distance↑, E/E’↓, cardiac collagen volume↓, CSA of cardiomyocytes↓	[153]
		aerial parts	ethyl acetate	H9c2 cells	cell viability↑, LDH↓, ROS↓, MDA↓, SOD↑, CAT↑, apoptotic rate↓, caspase-3↓, Bax↓, Bcl-2↑	[131]
		plant	70% ethanol	H9c2 cells	cell viability↑, pathological changes↓, CK-MB↓, LDH↓, AST↓, percentage of apoptotic cells↓, Bcl-2↑, Bax↓, cleaved-capase-3↓, cytochrome c↓, the phosphorylation of p38↓, activation of p38 MAPK↓	[154]
		aerial parts	caffeic acid tetramers	cardiomyocytes of neonatal rats	cell viability↑, apoptosis rate↓	[69]
		-	fraction containing flavonoids	animal model (rats)	LDH↓, CK-MB↓, MDA↓, SOD↑, infarct size↓, apoptotic index↓, mitochondrial injury↓, Bax↓, cleaved caspase-3↓, caspase-7↓, caspase-9↓, Bcl-2↑	[110]
		aerial parts	fraction containing flavonoids	animal model (rats)	The percent recovery of LVDP↑, HR↑ and CF↑, CK↓, LDH↓, infarct size↓, MDA↓, SOD↑, GSH/GSSG↓	[132]
		aerial parts	70% methanol	animal model (rats)	LVDP↓, HR↓, incidence of VT↓, VT duration↓, total VEBs↓, infarct size↓, volume of infarcted tissue↓	[155]
		plant	fraction containing flavonoids	animal model (rats)	Apo-A1↑ Apo-E↑, pulmonary artery pressure↓, no abnormalities in the cerebral parenchymal vessels.	[156]
		plant	methanol, water	animal model (rats)	SOD↑, GSH-Px↑, IL-6↓, CRP↓, MDA↓, pulmonary artery pressure↓,	[31]
	*D. tanguticum*	plant	luteolin-7-*O-β*-D-glucopyranoside	H9c2 cells	cell viability↑, CK↓, LDH↓, the morphological changes↓, Ca^2+^↓	[102]
		plant	compounds from 95% ethanolic extract	the rat aortic rings	endothelium-dependent vasodilatory effects on rat aortic rings↑, eNOS↑, NO↑	[83]
Treatment of metabolic diseases	*D. kotschyi*	leaves, flowers	different extracts	anti- pancreatic α-amylase	IC_50_ = 0.34–0.59 mg/mL (leaves), 1.72–2.67 mg/mL (flowers)	[14]
		aerial parts	50% ethanol extract	3T3-L1 adipocytes, animal model (rats)	MDA↓, SOD↑, POD↑, p-AKT↑, p-FOXO1↑, PPARγ↑, SREBP-1↑, p-JNK↓, level of water intake↓, lasting blood glucose↓, TG↓, TC↓, LDL↓, HDL↑, number of adipocytes↓, adipocyte area↑,	[157]
		leaves	70% methanol polyphenolic fraction	animal model (rats)	TG↓, TC↓, LDL↓, HDL↑, AI↓	[158]
	*D. polychaetum*	aerial parts	methanol	animal model (rats)	blood glucose↓, TG↓, TC↓, MDA↓, GSH↑, SOD↑, CAT↑, ALT↓, creatinine↓	[60]
	*D. subcapitatum*	aerial parts	70% ethanol	animal model (rats)	TG↓, TC↓, LDL↓, ALT↓, AST↓, blood glucose↓, hepatic steatosis↓, relative liver weight↓	[61]
	*D. tanguticum*	plant	phenylacetamide glycosides	3T3-L1 adipocytes	glucose consumption in adipocytes↑	[84]
Anticancer	*D. kotschyi*	aerial parts	ethanol	^(1)^ MCF-7 cells ^(2)^ HT-29	^(1)^ IC_50_ = 133 µg/mL, ^(2)^ IC_50_ = 126 µg/mL	[13]
		aerial parts and leaves	ethanol	HepG2 cells	cell proliferation↓	[159]
		aerial parts	different fractions, luteolin	^(1)^ Calu-6 and ^(2)^ Mehr-80 cells	^(1)^ IC_50_ = 79.1–168.75 μg/mL, ^(2)^ IC_50_ = 124.2–200.0 μg/mL (fractions), ^(1)^ IC_50_ = 56.32 μg/mL, ^(2)^ EC_50_ = 78.32 μg/mL (luteolin)	[160]
		aerial parts	different extracts	MDA-MB-23 cells	survival rate↓,	[130]
		aerial parts	50% ethanol	animal model (rats), isolated rat hepatocytes	hepatocytes of tumoral rats: MMP↓, mitochondrial ROS↑, mitochondrial swelling↑, cytochrome c release↑	[161]
		aerial parts	isolated flavonoids	HL-60, HT-29, AGS, SaOs-2, WEHI-164 cells	IC_50_ = 5.1–>50 µg/mL	[118]
		leaves	methanol, xanthomicrol	K562, HL60, KB, Hela, A549, Saos-2, A2780-CP, A2780-S, MCF-7, A375, A172 l cells, animal model (mice)	IC_50_ = 6.8–100.3 µg/mL (extract), IC_50_ = 0.88–1.69 µg/mL (xanthomicrol), tumor volume in mice↓	[162]
	*D. moldavica*	aerial parts	70% ethanol	DLD-1	IC_50_ = 0.40–0.54 μM GAE (for different cultivar)	[98]
		aerial parts	phenolic compounds	^(1)^ KMS-12-PE, ^(2)^ Molm13	^(1)^ IC_50_ = 25.65–>50 μM ^(2)^ IC_50_ = 21.74–>50 μM	[116]
		aerial parts	lignans	NCI-H292 cells	dracomolphin E: EC_50_ = 18.9 μM PARP↑, caspase-3↑, Bax↑, Bcl-2↓, Bcl-XL↓	[119]
		aerial parts	tilianin	FaDu cells	cell proliferation ↓ apoptosis↑, expression of TLR4↑, Bcl-2↓, Bcl-xL↓, Bad↑, Bax↑, cytochrome c↑, caspase-3↑, PARP↑, TNF-α↑, IL-6↑	[163]
	*D. palmatum*	leaves	different fractions	PC-3 cells	cell apoptosis↑, SOD-2↑, caspase-8↑, cleaved PARP↑, Bcl-2/Bax ratio↑, p-AKT signaling↓	[164]
		aerial parts	70% ethanol	DLBCL lines, animal model (mice)	IC_50_ = 235.0 and 624.6 μg/mL, cleaved PARP-1↑, caspase 3↑, caspase 3/7↑, Bcl-2↑, Bcl-xL↓, Mcl-1↓, Bax↑, Bak↑, Myc↓	[165]
	*D. peregrinum*	plant	diosmetin	U251 and U138 cell, animal model (mice)	proliferation↓, migration↓, invasiveness in vivo↓, apoptosis ratio↑, TGF-β1↓, E-cadherin↑, Bcl-2 expression↓, Bax↑, cleaved caspase-3↑, tumor growth↓	[54]
	*D. polychaetum*	aerial parts	ethanol	^(1)^ MCF-7, ^(2)^ HT-29 cells	^(1)^ IC_50_ = 90 µg/mL, ^(2)^ IC_50_ = 140 µg/mL	[13]
Antibacterial	*D. kotschyi*	leaves, flowers	different extracts	*Micrococcus luteus*, *Staphylococcus aureus*, *Escherichia coli*, *Pseudomonas aeruginosa*	MIC = 3.75–30 mg/mL, MBC = 15–30 mg/mL	[14]
		leaves	70% methanol	different *M. tuberculosis* strains	IC_50_ = 40–320 μg/mL	[65]
		aerial parts	different extracts	*Staphylococcus aureus*, *Salmonella enterica*, *Listeria monocytogenes*, *Bacillus cereus Enteric aerogenes*, *Escherichia coli*	MIC = 0.781–100 mg/mL, MBC = 1.562–>100 mg/mL	[166]
	*D. moldavica*	aerial parts	70% ethanol	*Staphylococcus aureus*, *Escherichia coli*, *Pseudomonas aureginosa*	MIC = 0.171–0.825 μM GAE/100 μL, MBC = 0.343–3.300 μM GAE/100 μL	[98]
		plant	ethyl acetate	*S. aureus* strain	MIC_50_ = 0.39–1.56 mg/mL, bacteria biofilm formation↓ (at 50–200 μg/mL), cell membrane integrity↓, cell size↓, cell lyse↑, changes in the bacterium protein profile	[167]
		leaves	ethanol	*E. coli*, *K. pneumonia*	MIC = 10–40 mg/mL	[168]
	*D. polychaetum*	aerial parts	no information	*S. aureus* strains	MIC = 0.781–25 mg/mL, MBC = 1.56–50 mg/mL, bacteria biofilm formation↓, expression of sar, bap, icaD, and icaA↓	[169]
Antifungial	*D. kotschyi*	leaves, flowers branches	methanol	*C. albicans*	MFC = 66.42–125 mg/mL, MIC = 59.77–112.5 mg/mL	[16]
	*D. multicaule*	flowering aerial parts	ethanol	*C. albicans*	MIC = 50 μg/mL, MFC > 50 μg/mL	[77]
Anti-protozoa	*D. kotschyi*	plant	isokamferide, apigenin	*Trypanosoma cruzi*	MLC = 30 µM isokamferide, MLC = 70 µM apigenin	[96]
	*D. subcapitatum*	aerial parts	isolated flavonoids	*Trypanosoma cruzi*	MLC = 30–>400 µM	[62]
Antiviral	*D. heterophyllum*	aerial parts	ethanol	Vero cells, animal model (HSV-2 infected mice)	HSV-2 RNA replication↓, attachment of HSV-2 to cell surface↓, HSV-2 infectivity↓, EC_50_ = 0.99 mg/mL, mortality of HSV-2 infected mice↓, mice survival time↑	[5]
	*D. tanguticum*	aerial parts	ethanol	Vero cells, animal model (HSV-2 infected mice)	HSV RNA replication↓, attachment of HSV-2 to cell surface↓, HSV-2 infectivity↓, EC_50_ = 1.43 mg/mL, mortality of HSV-2 infected mice↓, mice survival time↑	[5]
Anti-spasmotic and antidiarrhoeal	*D. kotschyi*	aerial parts	70% ethanol, flavonoid extratc	rabbit bladder strips contracted with ^(1)^ Ach, ^(2)^ KCl, or ^(3)^ EFS	inhibition bladder contractions: EC_50_ = ^(1)^ 190–243 µg/mL; ^(2)^ 53–102 µg/mL, ^(3)^ 105–170 µg/mL	[170]
		aerial parts	70% ethanol, ethyl acetate fraction, isolated flavonoids	animal model (mice)	small intestine transit inhibition (at con. 20 mg/kg): ethanolic extract 32%, ethyl acetate 90 %, luteolin and apigenin 90%, apigenin-4′-galacotside 45%	[171]
		aerial parts	70% ethanol, isolated flavonoids	contractions induce by ^(1)^ KCl and/or ^(2)^ ACh in rat isolated ileum	EC_50_ = ^(1)^ 41 µg/mL, ^(2)^ 133 µg/mL (extract), EC_50_ = ^(1)^ 57–80 µM, ^(2)^ 80 µM (compounds)	[172]
		aerial parts	ethyl acetate, apigenin, luteolin	rabbit trachea contractions induce by ^(1)^ KCl and/or ^(2)^ ACh	EC_50_ = ^(2)^ 340 µg/mL (extract), EC_50_ = ^(1)^ 65–66 µg/mL, ^(2)^ 80–90 µg/mL (compounds)	[106]
		aerial parts	70% ethanol, heksane, apigenin	animal model (mice) diarrhea induced by ^(1)^ castor oil, or ^(2)^ magnesium sulfate	diarrhea reduction (at concentration 10 mg/kg): ^(1)^ 60–67%, ^(2)^ 59–66%	[173]
Anti-aging	*D. moldavica*	aerial parts	30% ethanol	C2C12 cells, *Caenorhabditis elegans*, clinical study	AMPK↑, FOXO1↑, collagen levels↑, col-144 promoter↑, skin moisturization↑, skin elasticity↑	[174]

ABTS—free radical scavenging activity, AChE—acethylcholine esterase, ADH—alcohol dehydrogenase, AI—atherogenic index, ALT—alanine aminotransferase, AMPK—5′ adenosine monophosphate (AMP)—activated protein kinase, ANP—atrial natriuretic peptide, anti-CII IgG—anti-type II collagen specific IgG, Apo-A1 and Apo-E—polipoproteins A1 and E, AST—aspartate aminotransferase, b-MHC—monoclonal antibody, Bax—apoptotic factor, Bad—apoptotic factor, Bak—apoptotic factor, Bcl-2—apoptotic factor, BChE—butyrylcholine esterase, Bcl-xL—pro-survival protein, BDNF—brain-derived neurotrophic factor, BNP—brain natriuretic peptide, Br—Br^•^ radical scavenging activity, CAT—catalase, CDS—disease cumulative score, CIRI—cerebral ischemia–reperfusion injury, CK—creatine kinase, CK-MB—isoenzyme of creatine kinase, CLB—carotene bleaching assay, Col-I—transcryption factor, Cox2—cyclooxygenase 2, CREB—cAMP response element-binding protein, CRP—C-reactive protein, CSA—cross-sectional area of cardiomyocytes, DPPH—2,2-diphenyl-1-picrylhydrazyl assay, E/E’—ratio between mitral E wave and E’ wave, EFS—electrical field stimulation, EGFR—epidermal growth factor receptor, EM-SA—erythrocyte membrane stabilizing activity, ERK—extracellular signal-regulated kinase and cAMP response element-binding protein, Fe^2+^—ferrous ion scavenging activities, FOXO1—fork head box protein O-1, FN1—transcription factor, FRAP—ferric reducing antioxidant power, FST—forced swimming test, Gli1—transcription factor, GPX or GSH-px—gluthatione peroxidase, GSSG—oxided disulfide, GSTA—gluthatione S-transferase alpha, HBT—hole-board test, HDL—high density lipoprotein, HO-1—heme oxygenase, H_2_O_2—_hydroxyoxide radical scavenging activity, HR—the heart rate, HSV-2—herpes simplex virus type 2, ICAM-1—intercellular adhesion molecule-1, IFC—ipsilateral frontal cortex, IFNγ—interferon γ, IL-1β—interleukine 1β, IL-2—interleukine 2, IL-4—interleukine 4, IL-6—interleukine 6, IL-17a—interleukine 17a, iNOs—inducible nitricoxide synthase, Keap 1—klech like ECH-associated protein 1, LPO—anti-lipidperoxidation activity, LDH—lactate dehydrogenase, LDL—low density lipoprotein, LVDP—left ventricular developed pressure, Mcl-1—pro-survival protein, MFC—minimum fungicidal concentration, MDA—malondialdechyde, MBC—minimum bactericidal concentration, MIC—minimum inhibitory concentration MIRI—myocardial ischemia/reperfusion injury, MLC—minimal lethal concentration; MMP—mitochondrial membrane permeabilization, MTT—(4,5-dimethylthiazol-2-yl)-2,5-diphenyl tetrazolium bromide, NF-κB p65—nuclear factor kappa p65, NO—nitric oxide radical scavenging activity, NOX-4—NADPH oxidase 4, NQO-1—NAD(P)H—quinone dehydrogenase 1, Nrf_2_—nuclear factor erythroid 2, NT-3—neurotrophin 3, O_2_—superoxide radical scavenging, OH—hydroxy radical scavenging activity, 8-OHdG—8-hydroxyl-doxyguanoside, PARP—poly (ADP-ribose) polymerase, PARP-1—poly (ADP-ribose) polymerase 1, PF—pulmonary fibrosis, PCNA—proliferating cell nuclear antigen, PGC-1α—peroxisome proliferator-activated receptor γ coactivator-1α, PGE2—prostaglandin E_2_, p-JNK—c-Jun N-terminal kinase, PPARγ—peroxisome proliferator-activated receptor gamma, Ptch1—transcription factor, ROS—reactive oxygen species, SBP—systolic blood pressure, SOD—superoxide dismutase, α-SMA—transcription factor, SMO—transcription factor, Shh—transcription factor, SREP-1—sterol regulatory element binding protein—1, TBA—total bile acid, TC—total cholesterol, TG—triglycerides, TGF-*β*—transforming growth factor-β, TGF-*β*1—transforming growth factor-β1, TLR4—toll like receptor, TNF-*α*—tumor necrosis factor alpha, TST—tail suspension test, TUNEL—Terminal deoxynucleotidyl transferase dUTP nick end labeling, WDM—well diffusion method, VEBs—the total number of ventricular ectopic beats, (VCAM-1) of VSMCs—vascular cell adhesion molecule -1, VF—ventricular fibrillation, VT—the number of beats occurring as ventricular tachycardia, cell lines: A172, GBM—human glioblastoma, A2780-CP and A2780-S—human ovarian carcinoma resistant to cisplatin, A375—human malignant melanoma, A549, AGS—human gastric adenocarcinoma, ATCC CCL-221—human adenocarcinoma, BJ—fibroblasts cell lines, C2C12—murine myoblast cells, Caco-2—human carcinoma cells, Calu-6—human pulmonary adenocarcinoma, CD11b+ Gr1+ MDSCs—myeloid-derived suppressor cells, FaDu—human pharyngeal squamous carcinoma, H9c2—rat cardiomyoblast cells, HaCaT—human epidermal keratinocyte, Hela—human cervix carcinoma, HFFF-P16—human fetal foreskin fibroblast, HL60—human promyeloid leukemia, HRMC—human renal mesangial cells, HT-29—human colon carcinoma, K562—human chronic myeloid leukemia, KB—human oral epithelial carcinoma, KMS-12-PE—human myeloma, LO2 cells—human hepatic cells, MCF-7, MDA-MB-23—human breast adenocarcinoma, Mehr-80 and NCI-H292—human lung carcinoma, PBMNs—peripheral blood mononuclear cells, PC-3—human, prostate cancer, RAW 264.7—mouse-originated macrophages, Saos-2—human osteogenic sarcoma, U251, U138, T98, U-87—human glioma, VSMC—rat vascular smooth muscle cells, WEHI-164—murine fibrosarcoma.

### 6.1. Antioxidant Activity

During their metabolism, cells generate reactive oxygen species (ROS) or other free radicals. While a balance between ROS formation and inactivation is needed to ensure proper functioning [175], excessive ROS production and/or insufficient antioxidant defense results in oxidative stress. Fortunately, polyphenols are potent antioxidants with multiple mechanisms of action, which can effectively neutralize ROS by acting as free radical scavengers.

The most effective solvents for extracting *Dracocephalum* compounds responsible for antioxidant activity are methanol, ethanol, alcohol-water mixtures, and ethyl acetate. These extracts have typically been evaluated using in vitro assays (Table 3). The most common antioxidant test was the DPPH assay, though additional tests also assessed the potential of *Dracocephaulum* species to neutralize other reactive molecules (H_2_O_2_, ABTS, superoxide, hydroxyl, NO radicals), iron reduction, carotene bleaching, and lipid peroxidation (Table 3). Most studies focus on *D. moldavica*, analyzing whole aerial parts or its specific organs (leaves, flowers), and most are based on methanolic and ethanolic extracts.

A study based on comet assay and micronucleus test found *D. moldavica* extract to protect against bleomycin-induced genotoxicity in human dermal fibroblasts, most likely due to its antioxidant potential [68]. Also the DPPH scavenging potential of acacetin-7-*O*-(4”-acetyl)-glucopyranoside, isolated from *D. moldavica* shoots, has been evaluated [97]. In addition, *D. kotschyi* has been studied; while most research has focused on extracts of aerial parts, some studies have analyzed the leaves and flowers separately, revealing differences in antioxidant potential and plant age-related variations [127] (Table 3).

For *D. heterophyllum*, several studies indicate that its ethyl acetate extract has the greatest activity. Its antioxidant properties are believed to derive from its rosmarinic acid, acteoside, decaffeoylverbascoside, and 2′-acetylplantamajoside content, with IC₅₀ values ranging from 19.03 to 26.63 µM (DPPH assay) [88]. Dang et al. [87] indicate that four gingerols isolated from this species have antioxidant activity. Individual studies have also reported antioxidant activity in *D. polychaetum*, *D. foetidum*, *D. multicaule*, *D. palmatum*, *D. rupestre*, *D. ruyschiana*, and *D. tanguticum*, with most evaluating free radical scavenging potential and reducing power (Table 3). Additionally, eriodictyol-7-*O*-glucoside isolated from *D. rupestre* significantly improved the survival of human renal mesangial cells exposed to cisplatin; this was attributed to activation of the Nrf2 pathway, which boosts the cellular antioxidant defense system [80].

### 6.2. Anti-Inflammatory Properties

Inflammation is a well-known risk factor in the pathogenesis of inter alia infections, rheumatoid arthritis, autoimmune diseases, type 2 diabetes mellitus, and cancer [176]. Numerous in vitro and animal studies have indicated that the *Dracocephalum* genus may play a significant role in preventing or alleviating these ailments due to their anti-inflammatory properties.

Zheng et al. [138] and Shi et al. [126] report that various extract fractions from the aerial parts of *D. heterophyllum* protected against concanavalin A-induced liver injury (Table 3). The treatment improved the survival of mice by limiting hepatic damage and inflammatory infiltrates while reducing the number of apoptotic cells. Additionally, the ethyl acetate extract of *D. heterophyllum* demonstrated potential in treating non-alcoholic steatohepatitis and liver fibrosis in mouse models by reducing cholestasis and decreasing the inflammatory response. The data indicate that the rosmarinic acid present in the extract has multi-target therapeutic activity [46]. In addition, *D. tanguticum* extracts had a protective effect against alcoholic liver disease in vitro and in vivo, and its mechanism of action may be related to the activation of Nrf2/Keap-1 and inhibition of the P65/NF-κB signaling pathways [148]. Treatment with the ethyl acetate extract of *D. heterophyllum*, containing verbascoside, rosmarinic acid with its methyl ester, luteolin, diosmetin, and dehydrodipine-9-*β*-D-glucoside, significantly ameliorated IRBP-induced experimental autoimmune uveitis in mice by suppressing the proliferation of autoreactive, antigen-specific CD4⁺ T cells [120]. Other research indicated that *D. heterophyllum* extract may serve as an anti-inflammatory agent in collagen-induced arthritis in mice [137].

Xing et al. [146] examined the effects of total flavonoid extract from *D. moldavica* aerial parts on the proliferation, migration, and adhesion molecule expression of TNF-α-induced rat vascular smooth muscle cells suggesting its potential in the treatment of atherosclerosis (Table 3). The flavonoid extract from this species also improved lung function in bleomycin-induced pulmonary fibrosis in mice by reducing inflammatory factor levels [143]. The *D. moldavica* extract demonstrated anti-inflammatory and antiseptic effects in lipopolysaccharide (LPS)-induced RAW 264.7 macrophages; the treatment increased survival rate in mice with septic shock [145]. Furthermore, Gang et al. [142] found *D. moldavica* extract to have anti-colitis effects in a dextran sulfate sodium (DSS)-induced chronic colitis model, highlighting its ability to modulate gut microbiota and inflammatory pathways.

Studies also indicated that *D. moldavica* and *D. kotschyi* have therapeutic effects on acetic acid-induced ulcerative colitis in rats [140,144]. Moreover, the methanolic extract of *D. kotschyi* and its isolated compound calycopterin exhibited pro-apoptotic activity in lymphocytes, inhibited lymphocyte proliferation, and suppressed nitric oxide production and pro-inflammatory cytokine activity in LPS-stimulated mouse macrophages [66,141].

The acetone extract of *D. foetidum* and three compounds isolated from the species, viz. a melitric acid A derivative, acacetin malonyl glucoside and glucuronide, showed significant hyaluronidase inhibitory activity, surpassing that of disodium cromoglicate [7]. Another study demonstrated that the alcoholic extract of *D. rupestre* effectively ameliorated intestinal inflammation in *Salmonella*-infected mice, and its ethyl acetate fraction exhibited hepatoprotective effects against CCl_4_-induced hepatic injury [57,146]. Two studies reported that compounds isolated from *D. peregrinum* (peregrinumin A, B, and C, peregrinumcin A, and A1′-methyl-2′-hydroxyethyl ferulate) exhibited notable anti-inflammatory activity in LPS-activated macrophages by inhibition of nitric oxide production [94,112] (Table 3).

### 6.3. Neuroprotective and Neuromodulatory Activity

Cognitive decline induced by chronic cerebral hypoperfusion is the most common problem during aging. The n-butanol extract obtained from *D. tanguticum* whole plant was found to improve the neurological abnormalities by upregulating antioxidant activity and neurotrophic factor synthesis after ischemia insult; this was particularly apparent in the ipsilateral frontal cortex and hippocampus of rat brains [51]. Other research has also demonstrated that *D. multicaule* extract can influence cognitive impairment and hippocampal neurodegeneration induced by chronic cerebral hypoperfusion [59].

The neuroprotective effect of *D. moldavica* on cerebral ischemia/reperfusion injury may be associated with the presence of flavonoids, which can limit oxidative stress and apoptosis [151]. *D. moldavica* extract, containing flavonoids, also displayed a protective effect on astrocytes under oxidative stress conditions induced by H_2_O_2_ [150]. Additionally, it has been documented that ethanolic extract of *D. moldavica* leaves significantly attenuated scopolamine-induced memory impairment [149], and had an antidepressant effect [71]. Moreover, *D. moldavica* aqueous extract inhibited neuronal activity in the central nervous system: treatment resulted in sedative properties, decreased locomotor activity, and muscular relaxation in experimental animals; these sedative effects may be influenced by the flavone glycosides present in the extract, especially the acacetin and diosmetin derivatives. Shafiee-Kandjani et al. [152] showed *D. ruyschiana* extract combined with *Lavandula angustifolia* and sertraline to be a remarkably efficient herbal remedy for the treatment of mild to moderate depression.

Extracts from three Iranian *Dracocephalum* species (*D. kotschyi*, *D. multicaule*, D. *polychaetum*) have been found to inhibit acetyl cholinesterase, butyryl cholinesterase, and tyrosinase; this may support the search for natural candidates for treating cognitive disorders [128,134]. Tyrosinase may play a significant role in the neurotoxicity created by dopamine associated with neurodegeneration in Parkinson’s disease, while the inhibition of AChE and BChE could be a therapeutic strategy for Alzheimer’s disease, senile dementia, ataxia, and myasthenia gravis.

### 6.4. Cardioprotective, Anti-Hypertension Activity

Some *Dracocephalum* species exhibit protective effects on the heart following myocardial ischemia–reperfusion injury and offer promise for the prevention and treatment of heart failure (Table 3). Most experiments have focused on *D. moldavica* or its flavonoids [101,110,131,132,153,154], with one study documenting the cardioprotective potential of its caffeic acid tetramers [69]. The myocardial protective effects of *D. moldavica* products are attributed to their antioxidant, anti-inflammatory, and anti-apoptotic properties. The studies provide convincing evidence that pre-treatment with extracts can improve heart rate and coronary flow, modify left ventricular developed pressure, inhibit myocardial apoptosis, enhance cardiomyocyte viability, and reduce infarct size. Moreover, it has been reported that *D. moldavica* may play a crucial role in preventing ischemia-/reperfusion-induced injuries, such as arrhythmia and infarction [155]. Additionally, the ethyl acetate fraction of *D. moldavica*, whose main metabolites are rosmarinic acid and tilianin, has been shown to be effective in preventing hypertension by modulating miRNA levels, gut microbiota, and host metabolism [32]. Moreover, Maimaitiyiming et al. [31] and Maimaiti et al. [156] report that the flavonoid-rich extract of *D. moldavica* has potential therapeutic effects in chronic mountain sickness of rats. *D. moldavica* treatment improved antioxidant parameters, reduced pulmonary artery pressure, and slowed the pathological effects in heart and brain tissue. Plants are believed to reduce oxidative damage by improving energy metabolism, protein metabolism, and anti-oxidative capacity.

Among other *Dracocephalum* species containing flavonoids, *D. heterophyllum* extract increased the viability of hypertrophic cardiomyocytes induced by angiotensis II [45]. Furthermore, luteolin and its derivatives isolated from *D. tanguticum* played significant roles in protecting against doxycycline-induced cardiotoxicity [102]. The 95% ethanol extract of *D. tanguticum* was found to exhibit endothelium-dependent vasodilatory effects, suggesting some components may have potential as antihypertensive agents [83].

### 6.5. Treatment of Metabolic Diseases

*Dracocephalum* species are also used in traditional medicine in diseases associated with metabolic disorders such as diabetes and hyperlipidemia. There are reports available confirming this spectrum of activity. The extract of aerial parts from *D. kotschyi*, *D. polychaetum*, and *D. subcapitatum* significantly decreased blood triglyceride, total cholesterol, and LDL-cholesterol levels and significantly increased HDL-cholesterol level in rats with hyperlipidemia caused by a high-fat diet, dexamethasone, or streptozotocin (Table 3) [60,61,157,158]. These species also lowered blood sugar level, whereas administration of *D. kotschyi* extract also demonstrated adipogenic effects on 3T3-L1 cells [157]. Moreover, extracts from leaves and flowers of *D. kotschyi* containing caffeic acid inhibited pancreas α-amylase activity, an enzyme essential for carbohydrate digestion [14], while phenylacetamide glycosides isolated from *D. tanguticum* exhibited moderate anti-hyperglycemic activity [84].

### 6.6. Anticancer Activity

Some *Dracocephalum* species displayed cytotoxic activity against various cancer cell lines; the potency depended on the species, type of extract, and type of cancer cell (Table 3). Most research in this area focuses on *D. kotschyi*. The cytotoxic effects of extracts from this species have been reported for various cancer cell lines, including human breast cancer (MCF-7, MDA-MB-231), human colon cancer (HT-29), human pulmonary adenocarcinoma (Calu-6), human lung cancer (Mehr-80, A549), hepatocellular carcinoma (HCC), human chronic myeloid leukemia (K562), human promyelocytic leukemia (HL-60), human cervical carcinoma (HeLa), human oral epithelial carcinoma (KB), human osteogenic sarcoma (Saos-2), human ovarian carcinoma (A2780), human malignant melanoma (A375), and human glioblastoma (A172) [13,159,160,162].

Some studies have identified flavonoids to have the greatest anticancer potential in *D. kotschyi* [118,160,162]. Eight flavonoids isolated from *D. kotschyi* exhibited activity against human acute promyelocytic leukemia (HL-60), human colon carcinoma (HT-29), human gastric adenocarcinoma (AGS), human osteosarcoma (Saos-2), and murine fibrosarcoma (WEHI-164), with IC_50_ values ranging between 5.1 and 50 µg/mL. The strongest effects were observed for apigenin and luteolin, while the weakest were seen for calycopterins and naringenins, though efficacy varied depending on the cell line [118]. Some flavonoids demonstrated preferential antiproliferative activity against specific malignant cell lines.

In another study, xanthomicrol was identified as the most potent cytotoxic component of *D. kotschyi* leaf extract [162]. In addition to in vitro cytotoxicity, *D. kotschyi* leaf extract also exhibited antitumor effects in vivo. It reduced tumor proliferation and volume in mice inoculated with WEHI-164 (fibrosarcoma) cells, and hepatocellular carcinoma induced by diethylnitrosamine in rats. The extract promoted ROS formation and stimulated mitochondrial membrane permeabilization and swelling in rat hepatocytes, ultimately inducing apoptosis in tumor cells.

Some studies have investigated the anticancer activity of phenolic compounds from *D. moldavica*. Its ethanolic extract was found to be effective against the colorectal adenocarcinoma (DLD-1) cell line [98]. Most anticancer studies on this species focused on isolated compounds, with tilianin demonstrating anticancer properties in in vitro experiments on human pharyngeal squamous carcinoma cells (FaDu cells). Tilianin inhibited malignant cell growth, reduced colony formation, and induced apoptosis [163]. Additionally, the lignan dracomolphin E, obtained from the aerial parts of *D. moldavica*, inhibited the proliferation of lung cancer NCI-H292 cells with an EC50 of 18.9 μM [119]. Diosmetin was the most effective polyphenol against the myeloma cell line KMS-12-PEW (IC_50_ = 26 µM), while cirsimaritin and xanthomicrol exhibited the strongest effects on the AML cell line Molm-13 (IC_50_ = 21.74 µM and 27.98 µM, respectively) [116]. Diosmetin isolated from *D. peregrinum* reduced the tumorigenicity of U251 glioma cells and promoted their apoptosis in vivo [54].

The methanol extract and ethyl acetate fraction from *D. palmatum* significantly decreased the viability of PC-3 prostate cancer cells [164]. Also, the fractions of *D. palmatum* ethanolic extract exhibited cytotoxicity against multiple large B-cell lymphoma cell lines (DHL4, DHL6, Ly1, Ly8, Ly19, and HBL1), with IC_50_ values ranging from 235.0 to 624.6 µg/mL. The most effective fraction contained flavonoids [165]. In addition, the ethanol extract of *D. polychaetum* inhibited a human breast cancer cell line (MCF-7) with an IC_50_ of 140 µg/mL and a human colon cancer cell line (HT-29) with an IC_50_ of 90 µg/mL [13].

Several mechanisms have been proposed for the anticancer activity of *Dracocephalum* species. However, most are believed to concern the promotion of apoptosis through suppression of anti-apoptotic Bcl-2 family proteins, disruption of mitochondrial membrane potential, activation of the TLR4 signaling pathway, and inhibition of MYC.

### 6.7. Antipathogenic Activity

Several studies have shown that extracts from aerial parts of *Dracocephalum* species, rich in phenolic compounds, exhibit antibacterial activity. *D. kotschyi* has demonstrated variable antibacterial activity depending on the type of extract used and the bacterial species tested. The ethyl acetate extract exhibited the strongest antibacterial effect, with minimum inhibitory concentration (MIC) values ranging between 0.781 and 12.5 mg/mL for Gram-positive bacteria (*Staphylococcus aureus*, *Bacillus cereus*, and *Listeria monocytogenes*) and between 25 and 100 mg/mL for Gram-negative bacteria (*Salmonella enterica*, *Escherichia coli*, and *Enterobacter aerogenes*) [14]. In another study, the acetone extract of *D. kotschyi* exhibited similar antibacterial activity against Gram-positive (*S. aureus* and *Micrococcus luteus*) and Gram-negative (*E. coli* and *Pseudomonas aeruginosa*) bacteria, with an MIC of 3.75 mg/mL [166]. The sensitivity of *Mycobacterium tuberculosis* to *D. kotschyi* methanolic extract varied depending on the bacterial strain. MIC_50_, i.e., 50% growth inhibition, was achieved at an extract concentration of 40 μg/mL for antibiotic-sensitive strains, and 320 μg/mL for multidrug-resistant strains [65].

Three studies have investigated the antibacterial activity of *D. moldavica*. Hydroethanolic extracts from different cultivars exhibited antibacterial activity, with significant variations depending on the bacterial species: the extracts were active against methicillin-resistant *S. aureus* (MRSA) and Gram-negative *E. coli* but showed no activity against *P. aeruginosa* [98]. In another study, the ethanolic extract inhibited the growth of antibiotic-resistant strains of *E. coli* and *Klebsiella pneumoniae*, with MIC values of 10 and 40 mg/mL, respectively [168]. In contrast, the ethyl acetate fraction exhibited significant antibacterial activity against five Gram-positive bacteria (*S. aureus*, *S. epidermidis*, *S. haemolyticus*, *Enterococcus faecalis*, and *E. faecium*) in a dose-dependent manner [168]. However, no antimicrobial activity was observed against Gram-negative bacteria at concentrations up to 250 mg/mL. Detailed analyses revealed that the extract, containing rosmarinic acid and flavonoids, caused significant structural and functional changes in MRSA cells, including decreased membrane integrity, increased cell lysis, inhibition of biofilm formation, and alterations in the bacterial protein profile. Anti-MRSA properties were also observed for *D. polychaetum* extract [169], which inhibited bacterial growth (MIC = 0.781–25 mg/mL), suppressed biofilm formation, and downregulated the expression of biofilm-associated genes such as icaD, icaA, bap, and sar.

The ethanol extract of *D. multicaule* exhibited promising antifungal activity against *C. albicans*, with a MIC of 50 μg/mL [77]. Ghoratolhamide et al. [16] reported that the methanol extract of *D. kotshyi*, obtained under optimized conditions, inhibited the growth of *C. albicans*, with minimum fungicidal concentrations (MFC) and MICs of 66.42 mg/mL and 59.77 mg/mL, respectively. However, no antifungal activity against *Saccharomyces albicans*, *C. glabrata*, *C. krusei*, or *C. parapsilosis* was observed for different extracts of *D. moldavica* at concentrations up to 250 mg/mL [167].

Extracts of *D. heterophyllum* and *D. tanguticum* demonstrated activity against herpes simplex virus type 2 (HSV-2) in vitro and in vivo. These extracts inhibited HSV-2 infection primarily by reducing viral attachment to the cell surface, diminishing viral infectivity, and strongly suppressing HSV DNA replication at a concentration of 4 mg/mL. Additionally, the extracts of both species, administered at a dose of 1 g/kg per day, significantly prolonged the mean survival times and reduced the mortality of mice with HSV-2-induced encephalitis [5].

Interestingly, isokaempferide and apigenin isolated from the ethyl acetate extract of *D. kotschyi* and *D. subcapitatum* displayed trypanocidal activities against epimastigotes of *Trypanosoma cruzi*, the etiological agent of Chagas disease [62,96]. The minimum lethal concentrations of these compounds were 30 µM and 70 µM, respectively.

### 6.8. Other Activities

Some species of *Dracocephalum* have demonstrated spasmolytic activity. However, studies in this area have only been conducted on *D. kotschyi*. Extracts of *D. kotschyi* were shown to prevent contractions induced by acetylcholine, KCl, or electrical field stimulation of isolated rabbit bladder tissue [170] (Table 3). This suggests that the plant may be useful for managing urinary incontinence caused by involuntary bladder spasms. Moreover, *D. kotschyi* extract might be useful in bronchospastic conditions such as asthma and chronic bronchitis. The experiment demonstrated relaxant effects against KCl- and ACh-induced contraction of tracheal smooth muscle in rabbits, which could be attributed to the presence of flavonoids such as apigenin and luteolin [171]. Hydroalcoholic and hexane extracts of *D. kotschyi*, as well as isolated apigenin, reduced intestinal transit in mice with diarrhea induced by castor oil or magnesium sulfate administration. This confirms that *D. kotschyi* has antispasmodic and antidiarrheal properties in vivo, indicating its potential as a therapeutic agent for gastrointestinal disorders [173]. In addition, the hydroalcoholic extract of *D. kotschyi* inhibited contractions induced by both KCl and acetylcholine in rat ileum, with the effects mainly being attributed to apigenin and luteolin [172]. Moreover, the hydroalcoholic extract, its ethyl acetate fraction, and isolated compounds, including apigenin, luteolin, and apigenin-4′-galactoside, significantly inhibited small intestine transit in mice following oral administration of a charcoal meal [106]. In a subsequent stage, *D. kotschyi* extract was successfully formulated into standardized oral dosage forms, including capsules and syrup, for potential use in further clinical trials [17].

Wandrey et al. [174] report that the hydroethanolic extract of *D. moldavica* contains various flavonoids that activated the innate longevity pathway in mouse C2C12 cells via AMPK-FOXO1 signaling. Plant material also promoted collagen expression and increased collagen levels in the nematode *Caenorhabditis elegans*, an established model organism in aging research. Moreover, when used as a food supplement (DracoBelle™ Nu) daily for two months, it increased dermal and epidermal density, skin moisturization, and elasticity in subjects with sun-damaged skin [174].

## 7. Toxicity of *Dracocephalum* Species

So far, limited evidence is available on the toxicity of species within the genus *Dracocephalum*. Most such studies involve in vitro cytotoxicity assays using non-tumor human or animal cell lines. For instance, *D. palmatum* extract indicated no cytotoxicity on murine splenocytes and bone marrow cells, nor any significant impact on the viability of human peripheral blood mononuclear cells at concentrations up to 1 mg/mL [165]. Similarly, *D. kotschyi* extract yielded IC_50_ values of approximately 480, 690, and >1000 μg/mL against human lymphocytes at 24, 48, and 72 h, respectively [177]. Moreover, *D. moldavica* ethanolic extract exhibited no significant effect on foreskin fibroblast survival [98]. Moghaddam et al. [118] investigated the antiproliferative activity of flavonoids isolated from *D. kotschyi* against fetal foreskin fibroblasts. While hydroxyflavones such as luteolin and apigenin demonstrated cytotoxicity, methoxylated derivatives selectively inhibited malignant cell growth while sparing non-cancerous fibroblasts. Furthermore, a 48 h exposure to *D. moldavica* methanolic extract fractions, including those enriched with flavonoids, did not result in significant cytotoxicity in a L929 mouse fibroblast cell line (IC_50_ > 200 μg/mL) [160].

Only a few studies have investigated the potential toxicity of extracts and compounds derived from *Dracocephalum* species in in vivo animal models. Pouraboli et al. [60] report that the hydromethanolic extract of *D. polychaetum* was relatively safe at doses up to 1500 mg/kg, as no symptoms of toxicity or mortality were observed for 48 h following administration to rats. In addition, no significant alterations in tissue architecture or systemic toxicity were detected in the lungs, heart, liver, and kidneys of male nude mice injected intraperitoneally with *D. palmatum* extract at a dose of 50 mg/kg daily for three weeks [165]. *D. moldavica* ethanolic extract, administered orally at doses up to 5000 mg/kg, did not result in mortality in mice. However, mild adverse effects, including sedation and reduced locomotor activity, were observed at 750 mg/kg [71]. Conversely, the aqueous extract of *D. moldavica* exhibited toxicity when administered intraperitoneally, with an LD_50_ of 470 mg/kg [30]. The animals were monitored for signs of toxicity for one hour post-administration and subsequently every 24 h for seven days. At doses of 1000 mg/kg, adverse effects such as diarrhea, diaphoresis, piloerection, and ataxia appeared immediately and persisted for two hours until death. At 370 mg/kg, the animals exhibited decreased locomotor activity and flaccidity lasting 1–2 h, while at 600 mg/kg, complete mortality was observed.

The hydroalcoholic extract of *D. kotschyi* did not induce significant biochemical or hematological changes when administered orally to rats for 30 days at doses of 50, 100, or 200 mg/kg. However, the 200 mg/kg dose caused a slight increase in liver enzymes, while the lower dose increased creatinine levels. Histological examination revealed no tissue damage in the liver or kidneys [178].

Additionally, toxicity studies on xanthomicrol, a compound isolated from *D. kotschyi*, were conducted in mice. When the compound was administered intraperitoneally at doses of 40 or 50 mg/kg, no significant changes were observed in biochemical markers or histological analysis of the kidneys, intestines, lungs, or heart compared to controls [179].

In another study, Zhang et al. [5] assessed the acute toxicity of *D. heterophyllum* and *D. tanguticum* extracts following intraperitoneal injection in mice. The LD_50_ values were determined to be 14.99 g/kg for *D. heterophyllum* and 5.62 g/kg for *D. tanguticum*. Animals in the low-dose groups exhibited mild reactions, including transient listlessness and immobility for 3–5 min post-administration. In contrast, those receiving high doses experienced an initial excitation followed by coma, dyspnea, and impaired coordination.

Overall, available data indicate that most *Dracocephalum* species exhibit low acute toxicity, particularly when administered orally. However, for certain extracts, intraperitoneal administration may result in toxicity at higher doses. Additionally, while most *Dracocephalum* extracts are non-cytotoxic to normal mammalian cells, specific flavonoids may exert selective antiproliferative effects on malignant cells. Further studies are warranted to elucidate the long-term safety profile and potential organ-specific toxicities of these extracts.

## 8. Conclusions and Future Directions

A detailed review was performed of the current knowledge regarding the structures, activities, and pharmacology of phenolic compounds of *Dracocephalum* species. More than 300 bioactive phenols have been documented, with flavonoids and phenolic acids being the most prevalent forms. The most widely distributed flavonoids among *Dracocephalum* species are luteolin and apigenin derivatives, with high levels of mono-, di-, tri-, tetra-, and pentamethoxylated flavones. In addition, the predominant phenolic acids include chlorogenic acid, coumaric acid, rosmarinic acid, and their derivatives. Additionally, several studies highlight the anti-inflammation, neuroprotection, cardioprotection, hepatoprotection, and immuno-modulatory potential of the plants of the *Dracocephalum* genus and their metabolites. However, while in vitro and in vivo studies provide promising evidence of their pharmacological effects, critical gaps remain that hinder their integration into official medicine.

Most studies on phytochemical analysis of *Dracocephalum* species are qualitative in nature and do not take into account the level of production of bioactive compounds and the influence of environmental factors on their biosynthesis. However, it is the plant metabolites and their content that determine the pharmacological potential of the raw material; as such, future studies should include a determination of the levels of these bioactive metabolites, and standardize any raw materials that are used.

While it is important to identify the bioactive ingredients that may have therapeutic potential, it is also essential to ensure that they have adequate bioavailability. However, data from clinical trials regarding bioavailability, metabolism, pharmacokinetics, and standardized dosing *Dracocephalum* raw materials remain scarce. Future research should focus on the pharmacokinetics of *Dracocephalum*-derived compounds and the standardization of extracts; well-designed clinical trials are also needed to validate their therapeutic efficacy. Furthermore, the molecular mechanisms underlying pharmacological effects of *Dracocephalum* plants require deeper exploration through advanced biochemical approaches; interdisciplinary studies integrating metabolomics, genomics, and systems biology approaches are essential to fully elucidate their mechanisms of action and therapeutic potential. Addressing these gaps will be crucial for the potential development of *Dracocephalum* products as conventional pharmacological agents with standardized applications in modern phytotherapy.

## Figures and Tables

**Table 1:** Botanical description of the selected *Dracocephalum* species.

Species	Features						
	Habitat Form	Stem Form and Length (cm)	Leaf Lamina and Length	Number of Flowers in Verticillaster	Calyx Color and Length	Corolla Color and Length	References
*D. foetidum* Bunge.	annual	branches from the base, shoots ascending or prostrate (8–30 cm)	oblong or oblong-ovate, margin crenate (1–3 cm)	6	green (0.7–0.9 cm)	blue, blue-purple (about 3 cm)	[19]
*D. heterophyllum* Benth.	perennial	ascending or decumbent (10–15 cm)	blade broadly to narrowly ovate, base cordate, margin shallowly crenate or serrate (2.5 cm)	4–8	greenish (1.5–1.7 cm)	white (1.8–3.7 cm)	[20,21]
*D. kotschyi* Boiss.	perennial	erect (23–33 cm)	ovate-shaped (0.6–1 cm)	2–4	greenish (1.3–1.5 cm)	white or yellowish-white (1.9–2.8 cm)	[22,23]
*D. moldavica* L.	annual	erect or ascending (22–40 cm)	ovate-triangular, base cordate, margin remotely crenate, apex rounded (1.4–4 cm)	4	with purple veined (0.8–1 cm)	bluish purple (1.5–3 cm)	[20]
*D. multicaule* Montbr & Auch	perennial	(15–30 cm)	ovate or oblong-lanceolate, margin crenate (1.5–3–5 cm)	2–4	(1.5–1.7 cm)	cream or pale yellow, white (2.3–3.1 cm)	[24]
*D. palmatum* Steph. ax Willd.	perennial	ascending or decumbent (20–55 cm)	ovate-rounded (0.5–2 cm)	4–6	purple, (about 1 cm)	purple (2–3 cm)	[25,26,27]
*D. peregrinum* L.	perennial	erect (15–25 cm)	ovate-lanceolate to lanceolate, base cuneate (1.5–2.2 cm)	4–6	purple, (1–1.3 cm)	blue-purple, (2.2–2.8 cm)	[20]
*D. polychaetum* Bornm.	perennial	(10–20 cm)	oblong-lanceolate, crenate, serrate	2–4	greenish	yellowish	[28]
*D. rupestre* Hance.	perennial	ascending (15–42 cm)	triangular-ovate, base cordate, margin crenate-serrate (1.4–5.5 cm)	4–6	purplish (2–2.4 cm)	purple-blue, (3.8–4 cm)	[20]
*D. ruyschiana* L.	perennial	erect (20–60 cm)	linear to lanceolate-linear (3.4–6.2 cm)	4–6	(1.7–2.4 cm)	blue-purple (1.7–2.4 cm)	[20,27]
*D. subcapitatum* (Kuntze) Lipsky	perennial	erect or prostate (10–25 cm)	ovate or elliptical-ovate (1–1.5 cm)	6	(1.4–1.6 cm)	purple-blue to purple (2–3 cm)	[27]
*D. tanguticum* Maxim.	perennial	erect (to 55 cm)	blade pinnatisect, elliptic-ovate to elliptic, margin entire (2.6–7.5 cm)	2–6	purplish (1–1.4 cm)	purple-blue to dark purple (2–2.7 cm)	[20]

## Supplementary Materials

The following supporting information can be downloaded at: https://www.mdpi.com/article/10.3390/molecules30092017/s1. Table S1: Distribution of phenolic compounds in *Dracocephalum* species.
