# Supplementary material for: A Comprehensive Review of the Phenolic Compounds in Dracocephalum Genus (Lamiaceae) Related to Traditional Uses of the Species and Their Biological Activities
Source: Molecules. 2025 Apr 30;30(9):2017. doi: 10.3390/molecules30092017 (PMC12073305; doi:10.3390/molecules30092017)
Supplement: Supplementary file 1 [file molecules-30-02017-s001.zip › molecules-3586484-supplementary.pdf]

Table S1. Distribution of phenolic compounds in *Dracocephalum* species

No.	Compounds	*D. foetidum*	*D. hterophyllum*	*D. kotschui*	*D. moldavica*	*D. multicaule*	*D. palmatum*	*D. peregrinum*	*D. poluchaetum*	*D. rupestre*	*D. ruyschiana*	*D. subcapitatum*	*D. tanguticum*	References
1	2-hydroxybenzoic acid		+											[92]
2	4-hydroxybenzoic acid			+	+		+		+					[13,26,93]
3	3-hydroxybenzoic acid				+									[93]
4	3,4-dihydroxybenzoic acid				+									[93]
5	3-methoxy-4-hydroxybenzoic acid		+											[92]
6	2-hydroxy – 4-methoxy-3,6-dimethylbenzoic acid										+			[26]
7	gallic acid			+	+				+					[13,93]
8	methylgallic acid (methyl gallate)						+							[26]
9	*β*-glucogallin (1-galloyl-*β*-D-glucoside)						+							[26]
10	4-(*β*-D-glucolsylo)benzoic acid							+						[94]
11	methylparaben (methyl p-hydroxybenzoate)				+									[29]
12	ferulic acid (4-hydroxy-3-methoxycinnamic acid)			+	+			+	+		+			[10,13,42,94]
13	1′-methyl-2′-hydroxyethyl ferulate							+						[94]
14	*p*-coumaric acid (4-hydroxycinnamic acid)			+	+			+	+		+		+	[10,13,42,94,95]
15	methyl *trans-p*-coumaric acid										+			[42]
16	1-O-(4-coumaroyl)-glucoside										+			[26]
17	benzyl-2-*cis p*-coumaroyl-*α*-L-rhamnosyl(1→6)-*β*-D- glucoside										+			[42]
18	benzyl-2-*trans p*-coumaroyl-*α*-L-rhamnosyl(1→6)-*β*-D- glucoside										+			[42]
19	benzyl-4-*trans p*-coumaroyl-*α*-L-rhamnosyl(1→6)-*β*-D- glucoside										+			[42]
20	3-*p*-coumaroyl-5-caffeoylquinic										+			[42]
21	caffeic acid				+		+	+			+			[10,25,26,94]
22	caffeoyl-*β*-D-glucoside		+				+				+			[26,87]
23	caffeic ethyl ester (ethyl caffeate)						+							[26]
24	caffeic methyl ester (methyl caffeate)							+						[94]
25	caffeoylshikimic acid										+			[26]
26	chlorogenic acid (3-*O*-caffeoylquinic acid)			+	+		+	+	+		+			[10,13,25,42,94]
27	methyl chlorogenate												+	[95]
28	3,5-dicaffeoylquinic acid (izochlorogenic acid A)										+			[42]
29	3,4-dicaffeoylquinic acid (izochlorogenic acid B)										+			[42]
30	caftaric acid (caffeoyl tartaric acid)						+							[25]
31	cichoric acid						+							[25]
32	rosmarinic acid ((3,4-dihydroxycinnomoyl)-3-(3,4-dihydroxyphenyl)lactic acid)	+	+	+	+		+		+				+	[7,10,13,25,85,92,96]
33	methyl rosmarinate (3′-*O*-methyl rosmarinic acid)	+	+		+								+	[7,92,95,97]
34	ethyl rosmarinate		+		+									[92,97]
35	buthyl rosmarinate												+	[95]
36	sodium rosmarinate				+									[69]
37	rosmarinic acid glucoside (salviaflaside)	+			+									[7,69]
38	3-(6-synapoyl-*β*-D-glucosyl)-rosmarinic acid	+												[7]
39	3-(6-feruloyl-*β*-D-glucosyl)-rosmarinic acid	+												[7]
40	4-*O*-(8-isoferuloyl)-rosmarinic acid	+												[7]
41	3-(6-malonyl-*β*-D-glucosyl)-rosmarinic acid	+												[7]
42	caffeic acid trimer	+												[7]
43	rabdosiin				+									[69]
44	sodium rabdosiin				+									[69]
45	methyl rabdosiin				+									[69]
46	danshensu				+									[69]
47	ellagic acid				+						+			[26,98]
48	salvianolic acid B				+		+						+	[25,99]
49	salvianolic acid G						+							[26]
50	salvianic acid C						+							[26]
51	prolithospermic acid						+							[26]
52	chrysin 6-C-glucoside (5,7-dihydroxyflavone-6-*C*-glucoside)										+			[26]
53	chrysin glucuronide										+			[26]
54	7,4′-dihydroxyflavone				+									[101]
55	apigenin (5,7,4′-trihydroxyflavone)	+		+	+	+	+	+	+	+	+	+		[7,10,13,25,26,55,62,100,102,109]

	Compounds	*D. foetidum*	*D. hterophyllym*	*D. kotschui*	*D. moldavica*	*D. multicaule*	*D. palmatum*	*D. peregrinum*	*D. poluchaetum*	*D. rupestre*	*D. ruyschiana*	*D. subcapitatum*	*D. tanguticum*	References
56	apigenin 7-*O*-*β*-D-glucoside (comosin)			+	+	+	+			+	+		+	[10,11,25,26,55, 100,102]
57	apigenin-7-*O*-(6″-*O*-acetyl)-*β*-glucoside						+							[103]
58	apigenin-7-*O*-(6″-*O*-malonyl)-*β*-glucoside	+					+							[7,103]
59	apigenin-7-*O*-galactoside				+									[104]
60	apigenin-7-*O*-*β*-D-glucuronide	+			+						+			[7,26,105]
61	apigenin-7-*O*-rutinoside (isorhoifolin)		+				+							[53,92]
62	apigenin-7-*O*-β-D-glucosyl-(1→2)-*β*-D-glucosyl -(1→2)-[*α*-L-rhamnosyl-(1→6)]-*β*-D-glucoside										+			[42]
63	apigenin 8-C-pentoside-6-C-hexoside										+			[26]
64	apigenin 8-C-hecoside-6-C-pentoside										+			[26]
65	apigenin-4′-galactoside			+										[106]
66	apigenin-4′-*O*-*β*-D-glucoside			+										[96]
67	apigenin-5-*O*-*β*-D-glucoside					+	+							[26,100]
68	apigenin-7,4′- dimethyl ether						+							[26]
69	apigenin-7-sulfate										+			[26]
70	luteolin (5,7,3′,4′-tetrahydroxyflavone)		+	+	+	+	+	+	+	+	+	+	+	[26,53,55,62,92,94,96, 100,102,105,114]
71	luteolin-7-*O*-*β*-D-glucoside (cynaroside)			+	+	+	+	+		+			+	[26,55,94,96,100, 102,105]
72	luteolin-7-*O*-(6″-*O*-acetyl)-*β*-glucoside						+							[103]
73	luteolin-7-*O*-(6″-*O*-malonyl)-*ß*-glucoside						+							[103]
74	luteolin-7-*O*-(6''-feruloyl)-*β*-D-glucoside							+						[94]
75	luteolin-7-*O*-*β*-D-neohesperidoside				+									[105]
76	luteolin -7,4′-di-*O*-glucoside						+							[107]
77	luteolin-7-*O*-rutinoside (scolimoside)		+				+	+						[92,94,107]
78	luteolin 7-*O*-rutinoside-4′-*O*-glucoside (cynarotriside)						+							[107]
79	luteolin-7,4′-di-*O*-neohesperidoside						+							[107]
80	luteolin-7-*O*-neohesperidoside-4′-*O*-sophoroside						+							[107]
81	luteolin 7,4′-di-*O*-rutinoside (dracopalmaside)						+							[107]
82	luteolin 7-*O*-D-glucosyl-D-glucoside												+	[50]
83	luteolin 7-*O*-*α*-L-arabinosyl-(1→6)-*β*-D-glucoside												+	[50]
84	luteolin 7-*O*-*β*-D-xylosyl-(1→6)-*β*-D-glucoside												+	[50]
85	luteolin-7-*O*-*β*-D-glucuronide	+			+						+			[7,26,105]
86	luteolin 7-*O*-*β*-D-glucuronide ethyl ester												+	[102]
87	luteolin-7-methoxy-3'-*O*-(3''-*O*-acetyl)-*β*-D-glucuronic acid-6''-methyl ester												+	[108]
88	luteolin-3′-*O*-*β*-D-glucuronide			+										[96]
89	luteolin 5-*O*-*β*-D-glucoside		+											[92]
90	luteolin-4′-*O*-rutinoside						+							[107]
91	luteolin-4'-*O*-glucoside						+							[25]
92	plantaginin (scutellarein-7-O-glucoside)							+						[94]
93	acacetin (4′-*O*-methylated flavone)				+	+	+	+			+			[26,53,100,105,109]
94	acacetin-7-*O*-glucoside (tilianin, moldavoside)	+		+	+		+	+			+		+	[7,26,53,94,96,102, 105]
95	acacetin-7-*O*-(3″-acetyl)-*β*-D-glucoside				+									[97]
96	acacetin-7-*O*-(4″-acetyl)-*β*-D-glucoside				+									[97]
97	acacetin-7-*O*-(6′′-*O*-acetyl)-*β*-D-glucoside (agastachoside)				+		+							[103,105]
98	acacetin-7-*O*-(6″-malonyl)-*β*-D-glucoside	+			+		+							[7,101,103]
99	acacetin-7-*O*-(3″-*O*-malonyl)-*β*-D-glucoside	+												[7]
100	acacetin-7-*O*-(3″,6″-*O*-di-malonyl)-*β*-D-glucoside	+												[7]
101	acacetin-7-*O*-glucuronide	+			+						+			[7,26,105]
102	acacetin-7-*O*-(2″-*O*-malonyl)-*β*-D-glucuronide	+												[7]
103	acacetin-7-*O*-(2″-*O*-acetyl)-*β*-D-glucuronide	+			+									[7,110]
104	acacetin-7-*O*-rutinoside (linarin)	+	+				+	+						[7,53,94,111]
105	peregrinumin A (acacetin-7-O-(2″,3″-O-diacetyl)-rutinoside)							+						[112]
106	peregrinumin B (acacetin-7-O-(2″,4″-O-diacetyl)-rutinoside)							+						[112]

No.	Compounds	*D. foetidum*	*D. hterophyllym*	*D. kotschui*	*D. moldavica*	*D. multicaule*	*D. palmatum*	*D. peregrinum*	*D. poluchaetum*	*D. rupestre*	*D. ruyschiana*	*D. subcapitatum*	*D. tanguticum*	References
107	peregrinumin C (acacetin-7-O-(3″,4″-O-diacetyl)-rutinoside)							+						[112]
108	acacetin-7-*O*-neohesperidoside				+									[105]
109	acacetin-7-*O*-*β*-D-glucosyl-(1→2)-*β*-D-glucosyl-(1→2)-[*α*-L-rhamnosyl-(1→6)]-*β*-D-glucoside										+			[42]
110	acacetin-7-*O*-D-glucosyl-(1→2)-3″-*O*-acetyl-*β*-D-glucosyl -(1→2)-[*α*-L-rhamnosyl-(1→6)]-*β*-D-glucoside										+			[42]
111	acacetin-7-*O*-*β*-D-glucosyl-(1→2)-6″-*O*-acetyl-*β*-D-glucosyl-(1→2)-[*α*-L-rhamnosyl-(1→6)]-*β*-D-glucoside										+			[42]
112	acacetin-8-*C*-glucoside						+							[26]
113	acacetin-8-*C*-glucoside malonylated						+							[26]
114	acacetin-8-*C*-glucoside methylmalonylated						+							[26]
115	diosmetin (4′-*O*-methylated flavone)		+		+		+	+						[26,92,94,105]
116	diosmetin-7-*O*-glucoside		+								+		+	[26,50,111]
117	diosmetin-7-*O*-*β*-glucuronide	+			+									[7,105]
118	diosmetin-7-*O*-rutinoside		+											[111]
119	diosmetin-7-*O*-neohesperidoside				+									[105]
120	diosmetin 7-*O*-*β*-D-glucose-(1→6)-*β*-D-glucoside												+	[50]
121	diosmetin 7-*O*-*α*-L-arabinose-(1→6)-*β*-D-glucoside												+	[50]
122	diosmetin 7-*O*-*β*-D-xylose-(1→6)-*β*-D-glucoside												+	[50]
123	diosmetin-7-*O*-*β*-D-glucosyl-(1→2)-6-*O*-acetyl-*β*-D-glucosyl-(1→2)-[*α*-L-rhamnosyl-(1→6)]-*β*-D-glucoside										+			[42]
124	diosmetin-7-*O*-*β*-D-glucosyl-(1→2)-*β*-D-glucosyl-(1→2)-[*α*-L-rhamnosyl-(1→6)]-*β*-D-glucoside										+			[42]
125	dracocephaloside A				+									[101]
126	geraniol-7-O- *β*-D-glucuronide (5,3′-dihydroxy-4′-methoxyflavone-7-O-glucuronide)				+									[101]
127	genkwanin (5,4'-dihydroxy-7-methoxyflavone, sakuranetin)			+		+	+							[53,96,100]
128	chrysoeriol (5,7,4′-trihydroxy-3′-methoxyflavone)				+		+							[53,113]
129	pedalitin (5,6,3',4'-tetrahydroxy-7-methoxyflavone)												+	[12]
130	pedalitin-3′-*O*-glucoside												+	[102]
131	pedalitin-6-*O*-glucoside (pedaliin)												+	[12]
132	pedalin-6′′-acetate												+	[12]
133	5,6,4'-trihydroxy-7-methoxyflavone (scutellarein 7-*O*-methyl ether, sorbifolin)												+	[102]
134	sorbifolin-6-*O*-glucoside (ladanetin-6-*O*-*β*-D-glucoside)												+	[102]
135	ladanetin-6-*O*-*β*-(6″-*O*-acetyl)glucoside												+	[102]
136	negletein (5,6-dihydroxy-7-methoxyflavone)						+							[26]
137	cirsimaritin (scrophulein, 5,4′-dihydroxy-6,7-dimethoxyflavone)			+	+	+			+			+		[11,100,105,114]
138	5,4'-dihydroxy-6,7-dimethoxyflavone 4'-*O*-*β*-D-glucoside												+	[95]
139	cirsiliol-4'-glucoside (4'-glucoside of 5,3',4'-trihydroxy-6,7-dimethoxyflavone)												+	[115]
140	5,7,4'-trihydroxy-3',5'-dimethoxyflavone												+	[95]
141	5,3',4'-trihydroxy-6,7-dimethoxyflavone												+	[95]
142	isothymusine (5,8,4'-trihydroxy-6,7-dimethoxyflavone)						+							[53]
143	pectolaringenin (5,7-dihydroxy-6,4'-dimethoxyflavone)												+	[108]
144	xanthomicrol (5,4'-dihydroxy-6,7,8-trimethoxyflavone)			+	+	+			+			+		[62,96,100,114,116]
145	nevadesin (5,7-dihydroxy-6,8,4′-trimethoxyflavone)						+							[26]
146	salvigenin (5-hydroxy-6,7,4′-trimethoxylflavone)				+		+	+						[53,105,109]
147	8-hydroxy-salvigenin (5,8-dihydroxy-6,7,4'-trimethoxyflavone)				+									[104]
148	circlineol (5,4'-dihydroxy-7,8,3′-trimethoxyflavone)			+		+			+			+		[100,114]
149	santaflavone (5-desmethylsinensetin, 5-hydroxy-6,7,4′,3′-tetramethoxyflavone)			+	+	+		+	+			+		[104,109,114]
150	gardenin B (5-hydroxy-6,7,8,4'-tetraamethoxyflavone)				+	+								[100,110]
151	gardenin A (5-hydroxy-6,7,8, 3',4',5'-hexamethoxyflavone)				+									[104]
152	isokaempferide			+		+						+		[62,96,100]
153	kumatakenin (5,4'-dihydroxy-3,7-dimethoxyflavone)					+								[100]
154	5,7,4′-trihydroxy-3,3′-dimethoxyflavone			+		+			+			+		[100,114]
155	5,3′,4′-trihydroxy-3,7-dimethoxyflavone							+						[94]

No.	Compounds	*D. foetidum*	*D. hterophyllum*	*D. kotschui*	*D. moldavica*	*D. multicaule*	*D. palmatum*	*D. peregrinum*	*D. poluchaetum*	*D. rupestre*	*D. ruyschiana*	*D. subcapitatum*	*D. tanguticum*	References
156	ermanin (5,7-dihydroxy-3,4′-dimethoxyflavone)			+					+			+		[100,114]
157	penduletin (5,4′-dihydroxy 3,6,7-trimethoxyflavone)			+		+								[96,100]
158	chrysosoplenetin (5,4'-dihydroxy-3,6,7,3'-tetramethoxyflavone)		+											[111]
159	calycopterin (5,4′-dihydroxy-3,6,7,8-tetramethoxyflavone)			+		+			+			+		[62,96,100,114]
160	4′-methylcalicopterin					+								[100]
161	3-hydroxyflavone				+									[104]
162	gossypetin (5,7,8,3′4′-pentahydroxyflavone)-3-*O*-rhamnosyl-(1→6)-glucoside		+											[12]
163	kaempferol		+		+		+	+			+			[26,94,105,111]
164	quercetin		+	+	+			+	+					[10,12,13,94,111]
165	kaempferol-7-*O*-glucoside				+									[97]
166	kaempferol-3-*O*-glucoside (astragalin)				+		+	+			+			[26,42,94,97]
167	kaempferol-3-*O*-rhamnoside							+						[94]
168	kaempferol-3-*O*-glucuronide										+			[26]
169	kaempferol-3-*O*-rutinoside						+							[26]
170	2′′-p-coumaryl astragalin				+									[12]
171	kaempferol-3-*O*-*β*-D-(6′′-*O*-*p*-coumaroyl)-galactoside				+									[12]
172	kaempferide-3-*O*-rhamnoside							+						[94]
173	quercetin-7-*O*-*β*-D-glucoside							+						[94]
174	quercetin-3-*O*-*β*-D-glucoside				+			+			+			[42,94,97]
175	quercetin-3-*O*-*β*-D-rhamnoside (quercitrin)				+			+						[93,94]
176	quercetin-3-*O*-galactoside				+									[105]
177	quercetin-3-*O*-rutinoside												+	[117]
178	quercetin 3-*O*-*β*-D-glucuronide										+			[42]
179	isorhamnetin (5,7,3,4′ -tetrahydroxy-3′-methoxyflavone				+									[12]
180	eriodictyol (5,7,3′,4′-tetrahydroxyflavan-4-one)						+	+		+			+	[23,55,94,102]
181	eriodictyol-7-*O*-*β*-D-glucoside (pyracanthoside, miscanthoside)						+			+	+		+	[25,26,55,102]
182	eriodictyol-7-*O*-(4′′-*O*-malonyl)-*β*-D-glucoside (pyrcanthoside 4′′-malonate)						+							[103]
183	eriodictyol-*O*-rutinoside						+							[53]
184	eriodictyol-7-*O*-(6′′-*O*-malonyl)-*β*-D-glucoside (pyrcanthoside-6′′-malonate)						+							[103]
185	naringenin (5,7,4′-trihydroxyflavanone)			+	+		+		+	+			+	[13,25,55,98,102,118]
186	prunin (naringenin-7-*O*-*β*-D-glucoside)						+			+	+		+	[25,26,55,102]
187	narirutin (7-*α*-L-rhamnoside-(1→6)-*β*-D-glucosylo-4′,5,7-trihydroxyflavan-4-one)												+	[99]
188	5,7,3′,5′-tetrahydroxyflavanone												+	[102]
189	5,7,3′,5′-tetrahydroxyflavanone-7-O-*β*-D-glucoside												+	[102]
190	dihydroquercetin (3,5,7,3′,4′-pentahydroxyflavan-4-one)						+							[26]
191	dihydrokaempferol (3,5,7,4′-tetrahydroxyflavan-4-one)						+							[26]
192	ampelopsin (3,5,7,3′,4′,5′-hexahydroxyflavan-4-one)						+							[26]
193	fustin (3,7,3′,4′-tetrahydroxyflavan-4-one)										+			[26]
194	(epi)catechin			+	+				+		+			[13,26,93]
195	gallocatechin						+							[26]
196	pelargonidin-3-O-glucoside-3-O-glucoside(callistephin)						+				+			[26]
197	cyanidin O-pentoside						+							[26]
198	cyanidin-3-O-glucoside (kuromarin)						+							[26]
199	cyanidin 3-O-(6′′-malonyl)glucoside						+							[26]
200	cyanidin 3-(acetyl)hexoside						+							[26]
201	cyanidin 3-O-(coumaroyl)hexoside						+							[26]
202	7-O-methyl-delphinidin-3-O-(2′′galloyl)-galactoside-3-O-(2′′galloyl)-galactoside)						+							[26]
203	peonidin O-pentoside						+							[26]
204	peonidin-3-O-glucoside						+							[26]
205	petunidin						+				+			[26]
206	procyanidin A-type dimer (epicatechin-catechin dimer)										+			[26]

No.	Compounds	*D. foetidum*	*D. hterophyllym*	*D. kotschui*	*D. moldavica*	*D. multicaule*	*D. palmatum*	*D. peregrinum*	*D. poluchaetum*	*D. rupestre*	*D. ruyschiana*	*D. subcapitatum*	*D. tanguticum*	References
207	alaschanisoside A				+									[29]
208	akequintoside A				+									[29]
209	7,8-dihydrodehydrodiconiferyl alcohol 4-*O*-*β*-D-glucoside				+									[29]
210	7,8-dehydrodiconiferyl alcohol 9′-*O*-*β*-D-glucoside				+									[29]
211	(7,8)-3′-demethyl-dehydrodiconiferyl alcohol 3′-*O*-*β*-D-glucoside				+									[29]
212	(+)-pinoresinol				+									[29]
213	(+)-pinoresinol *O*-*β*-D-glucoside		+											[12]
214	(+)-pinoresinol di-*O*-*β*-D-glucoside		+											[12]
215	syringaresinol				+									[12]
216	syringaresinol 4-*O*-*β*-D-glucoside		+		+								+	[12]
217	syringaresinol 4,4′-*O*-*β*-D-di-glucoside				+									[12]
218	hedyotisol A				+									[29]
219	hedyotisol B				+									[29]
220	oresbiusin B		+		+									[12,119]
221	dracomolphin A				+									[119]
222	dracomolphin B				+									[119]
223	dracomolphin C				+									[119]
224	dracomolphin D				+									[119]
225	dracomolphin E				+									[119]
226	3-(4-hydroxy-3-methoxyphenyl)-2-{2-methoxy-4[-3-methoxy-3-oxoprop-1-enyl]phenoxy}-prop-2-enoate				+									[119]
227	3-benzofurancarboxylic acid-2-(3,4-dihydroxyphenyl)-7-hydroxy-5-(3-methoxy-3-oxo-1-propenyl)-methyl ester				+									[119]
228	3-[2-(3,4-dihydroxyhenyl)-7-hydroxy-3-methoxycarbonyl-2,3-dihydro-1-benzofuran-5-yl]prop-2-enoate				+									[119]
229	(+)-piperitol				+									[116]
230	(9+)-9*α*-hydroxysesamin				+									[116]
231	hinokinin										+			[26]
232	dimethyl-secoisolariciresinol (2,3-dimethyl-2,3-di(4-hydroxy-3-methoxybenzyl)-1,4-butanediol						+				+			[26]
233	dehydrodipine-9-*β*-D-glucoside		+											[120]
234	(2S,3R)-2,3-dihydro-7-hydroxy-2-(4'-hydroxy-3'-methoxyphenyl)-3-hydroxymethyl-5-benzofuranpropanol-4'-*O*-*β*-D-glucoside												+	[83]
235	ferruginoside B		+											[87]
236	verbascoside (acteoside)		+											[87]
237	2′-*O*-acetylplantamaioside		+											[87]
238	decaffeoylverbascoside		+											[88]
239	3-phenylethyl-*β*-D-glucoside												+	[115]
240	osmanthuside F												+	[83]
241	hyuganoside IIIa												+	[83]
242	dracomolphesin A				+									[121]
243	dracomolphesin B				+									[121]
244	dracomolphesin C				+									[121]
245	dracomolphesin D				+									[121]
246	dracomolphesin E				+									[121]
247	dratanguticumide A												+	[84]
248	dratanguticumide B												+	[84]
249	dratanguticumide C												+	[84]
250	dratanguticumide D												+	[83]
251	dratanguticumide G												+	[83]
252	dratanguticumide H												+	[83]
253	dracocephins A									+				[122]
254	dracocephins B									+				[122]
255	dracocephins C									+				[122]
256	dracocephins D									+				[122]

No.	Compounds	*D. foetidum*	*D. hterophyllym*	*D. kotschui*	*D. moldavica*	*D. multicaule*	*D. palmatum*	*D. peregrinum*	*D. poluchaetum*	*D. rupestre*	*D. ruyschiana*	*D. subcapitatum*	*D. tanguticum*	References
257	drahebephins A		+											[92]
258	drahebephins B		+											[92]
259	drahebenine C		+											[92]
260	pinosylvin										+			[26]
261	resveratrol										+			[26]
262	5-benzyl alkohol glucoside										+			[42]
263	sibiricin A		+											[87]
264	5-methoxy-6-gingerol		+											[123]
265	6-shogaol		+											[123]
266	6-paradol		+											[123]
267	diacetoxy-6-gingeridol		+											[123]
268	arbutin						+							[53]
269	*O*-malonylarbutin				+		+							[30,53]
270	umbeliferone						+							[25]
271	aesculetin						+							[25]
272	prunasin							+						[112]
273	peregrinumcin A							+						[112]
274	isomethylfuranopinarine		+											[124]
275	demethylfuropriranine		+											[124]
276	alloimperatorin		+											[124]
277	alloisoimperatorin		+											[124]
278	citrusin C				+						+			[29,42]
279	4-allyl-1[(*β*-D-glucosyl)oxy]-2-hydroxybenzene				+									[29]
280	3,5-dihydroxyphenethyl alcohol 3-*O*-*β*-D-glucoside				+									[29]
281	3-hydroxy-2-[4-(3-hydroxy-1-propen-1-yl)-2-methoxyphenoxy]propyl *β*-D-glucoside				+									[29]
282	guaiacyl glycerol-3'-*O*-*β*-D-glucoside												+	[83]
283	benzyl benzoate-2,6-di-*O*-*β*-D-glucoside												+	[83]
284	benzyl-*β*-D-glucoside,												+	[108]
285	benzyl-*O*-*β*-D-xylosyl-(1→6)-*β*-D-glucoside												+	[83]
286	benzyl-6-[(2*E*)-2-butenoate]-*β*-D-glucoside												+	[108]
287	2-methoxy-4-(2-propen-1-yl)phenyl-*β*-D-glucoside												+	[108]
288	2-methoxy-4-(2-propen-1-yl)phenyl-6-acetate-*β*-D-glucoside												+	[108]
289	2-methoxy-4-(2-propen-1-yl)phenyl-6-[(2*E*)-2-butenoate]-*β*-D-glucoside												+	[108]
290	[(benzoxy)methyl]phenyl-4-*O*-*α*-L-rhamosyl(1→6)-*O*-*β*-D-glucoside										+			[42]
291	[3-hydroxy-3-methoxyglutaryl]methyl]phenyl-4-*O*-*β*-D-glucoside										+			[42]
292	dracompresin									+				[125]
293	tazettoside												+	[115]
